# Predicting nonpoint stormwater runoff quality from land use

**DOI:** 10.1371/journal.pone.0196782

**Published:** 2018-05-09

**Authors:** Brik R. Zivkovich, David C. Mays

**Affiliations:** University of Colorado Denver, Department of Civil Engineering, Denver, Colorado, United States of America; University of Georgia, UNITED STATES

## Abstract

Evaluating the impact of urban development on natural ecosystem processes has become an increasingly complex task for planners, environmental scientists, and engineers. As the built environment continues to grow, unregulated nonpoint pollutants from increased human activity and large-scale development severely stress urban streams and lakes resulting in their currently impaired or degraded state. In response, integrated water quality management programs have been adopted to address these unregulated nonpoint pollutants by utilizing best management practices (BMPs) that treat runoff as close to the source as possible. Knowing where to install effective BMPs is no trivial task, considering budget constraints and the spatially extensive nature of nonpoint stormwater runoff. Accordingly, this paper presents an initial, straightforward and cost-effective methodology to identify critical nonpoint pollutant source watersheds through correlation of water quality with land use. Through an illustrative application to metropolitan Denver, Colorado, it is shown how this method can be used to aid stormwater professionals to evaluate and specify retrofit locations in need of water quality treatment features reduce, capture and treat stormwater runoff prior to entering receiving waters.

## 1. Introduction

Although stormwater has long been regarded as a major factor in flooding of urban areas, only since the 1980s have planners, environmental scientists, and engineers recognized the additional role that stormwater plays in water quality impairment of urban watersheds and natural ecosystems [1]. Since the creation of the Clean Water Act in 1972, the U.S. Environmental Protection Agency (U.S. EPA) has continued to develop and establish new regulations and remediation practices to address discharges of pollutants into streams and rivers, which have led designers to focus on pollutants located in and sourced by urban stormwater runoff [2,3].

Urban populations have grown significantly since of creation of the Clean Water Act, and consequently stormwater pollutants have become the primary cause of impairment for urban surface waters [4,5,6]. In response to these impairments, watershed management practices have become a critical objective for quality improvement of streams and lakes [7,8]. Research provides strong evidence that urbanization creates excess sediments, nutrients, and metals that directly impact ecological properties and stability of surface waters [9,10,11,12,13]. These impacts create biogeochemical instabilities as new pathways are created for sediments to be washed into receiving streams and lakes during storm events [14,15].

Under Section 402 of the Clean Water Act, the National Pollutant Discharge Elimination System was adopted to set effluent-based standards for point pollution sources. Municipal Separate Storm Sewer System permits were developed to address additional gaps, in particular nonpoint sources that leave rivers and lakes prone to unregulated pollutants. Preliminary data summaries have been reported to characterize stormwater data collected under National Pollutant Discharge Elimination System permits for more than 200 municipalities throughout the country [16]. Additionally, under Sections 303(d) and 305(b) of the Clean Water Act, the U.S. EPA requests states to report water quality through National Water Quality Assessment Reports. These bi-annual integrated reports are used to assess streams and surface waters, identify impaired waters and their causes, and track the status of actions being taken to restore impaired waters. For example, using a recent Colorado Water Quality Assessment Report from 2010, approximately 19% of assessed streams and 49% of assessed lakes are impaired, which triggers the requirement for a total maximum daily load plan [17,18,19]. Total maximum daily load plans, in turn, typically comprise one or multiple best management practices (BMPs) to reduce runoff to required standards.

Many cities in the United States and elsewhere have adopted green infrastructure programs using low impact development (LID) to address nonpoint stormwater runoff [4,20,21,22,23]. LID designs, such as water quality detention basins, rain gardens, constructed wetlands, and grass swales, treat nonpoint runoff using natural hydrologic processes [3,24], such that LID designs act as buffers for reducing pollutants that reach streams [25,26]. With proper planning, design, and maintenance, these LID systems can effectively treat urban runoff and reduce the mass of pollutants that enter urban streams and lakes.

The motivation for this paper is that application of LID designs or specification of BMPs takes place under significant budget constraints, which limit the number of sites at which these interventions can be deployed. Perhaps even more important is that impaired rivers and lakes often have unknown sources of contamination. Accordingly, there is a need to identify a simple methodology, based on freely available information that can be used to identify and prioritize sites for nonpoint stormwater interventions [27]. Moreover, the variability of nonpoint urban water quality runoff with respect to different land uses, geographical locations, imperviousness, rainfall, and sampling procedures can make selection of appropriate sampling locations a challenging feat [10,28,29]. This study investigates the hypothesis that stormwater runoff quality can be predicted from land use data, using a regression approach calibrated with locally collected concentration measurements. There are several considerations motivating this focus on land use.

First, the correlation between land use and runoff water quality is well-documented [30,31,32,33]. By assuming that at least one solute concentration differs by land use, one can predict variability in runoff water quality form variability in land use. These studies show how built environment land use types and land use changes affect surface water quality. Of these models and approaches, the most widely accepted is the U.S. Department of Agriculture’s Agricultural Non-Point Source Pollution Model, which evaluates management decisions within the agricultural setting [31]. The basis of the Agricultural Non-Point Source Pollution Model lies within its set of modeling modules that have become a powerful tool for evaluating sediment loading, chemical loading, and water quality in large watersheds through various agricultural runoff datasets. Other approaches have shown promise using land use regression models to evaluate contaminant variability across different spatial regions, developing sediment and nutrient transport models, and evaluating land use types with such applications to spatial nutrient variability in groundwater [29,34,35,36]. Taken together, each of these studies has the same motivation: to promote more accurate measurements that can help aid water resources management decisions for any studied region anthropogenically affected.

There are two other motivations for focusing on land use. The second motivation is that land use data are spatially extensive, with databases providing complete coverage of every major metropolitan area in the United States. And third, land use data are freely available, which makes land use a practical source for resource-limited stormwater management agencies. Accordingly, the methodology presented in the present study can be used to aid stormwater professionals when locating retrofits of water quality features within urban areas that can be used reduce, capture and treat stormwater runoff prior to entering the surrounding natural streams and river systems. Additionally, this methodology can aid regulatory agencies and other interested parties in finding locations that are key sources of degradation in urban drainage systems.

This paper is organized as follows. After identifying relevant data on stormwater runoff quality and land use, the methodology to predict runoff concentrations from land use is presented. In brief, this methodology predicts concentrations of sediments, nutrients, and metals in nonpoint stormwater runoff as a function of land use broken down into the three broad developed land use categories of residential, commercial, and other. Results are presented to summarize concentration data, display land use data, and predict stormwater runoff quality. Validation is provided for four mixed land use sampling locations not used in model development, and then an illustrative example is provided for a particular catchment in Denver, Colorado. The paper concludes with a discussion of the strengths and limitations of this methodology and concludes with a recapitulation of the methodology presented.

The primary objective of this paper is to offer a preliminary assessment tool to evaluate pollutant loading distribution in a watershed. Using freely available data, if at least one measured solute concentrations differs consistently across different land use categories then this parsimonious approach allows for prediction of stormwater runoff quality from correlation with land use classification. Taking advantage of well-documented monitoring practices, this new geospatial assessment approach presents a first-layer, cost-effective way to identify nonpoint stormwater areas of concern within a watershed. Most importantly, the universal concept behind the tool allows it to be applied across all scales within a delineated watershed (i.e. scaling to a block draining to an inlet). As a result, the time and effort required for LID planning and identification of nonpoint sources can be reduced, as this approach identifies and standardizes these nonpoint source areas contribution of pollutants in stormwater runoff.

## 2. Methods

**Fig 1:**
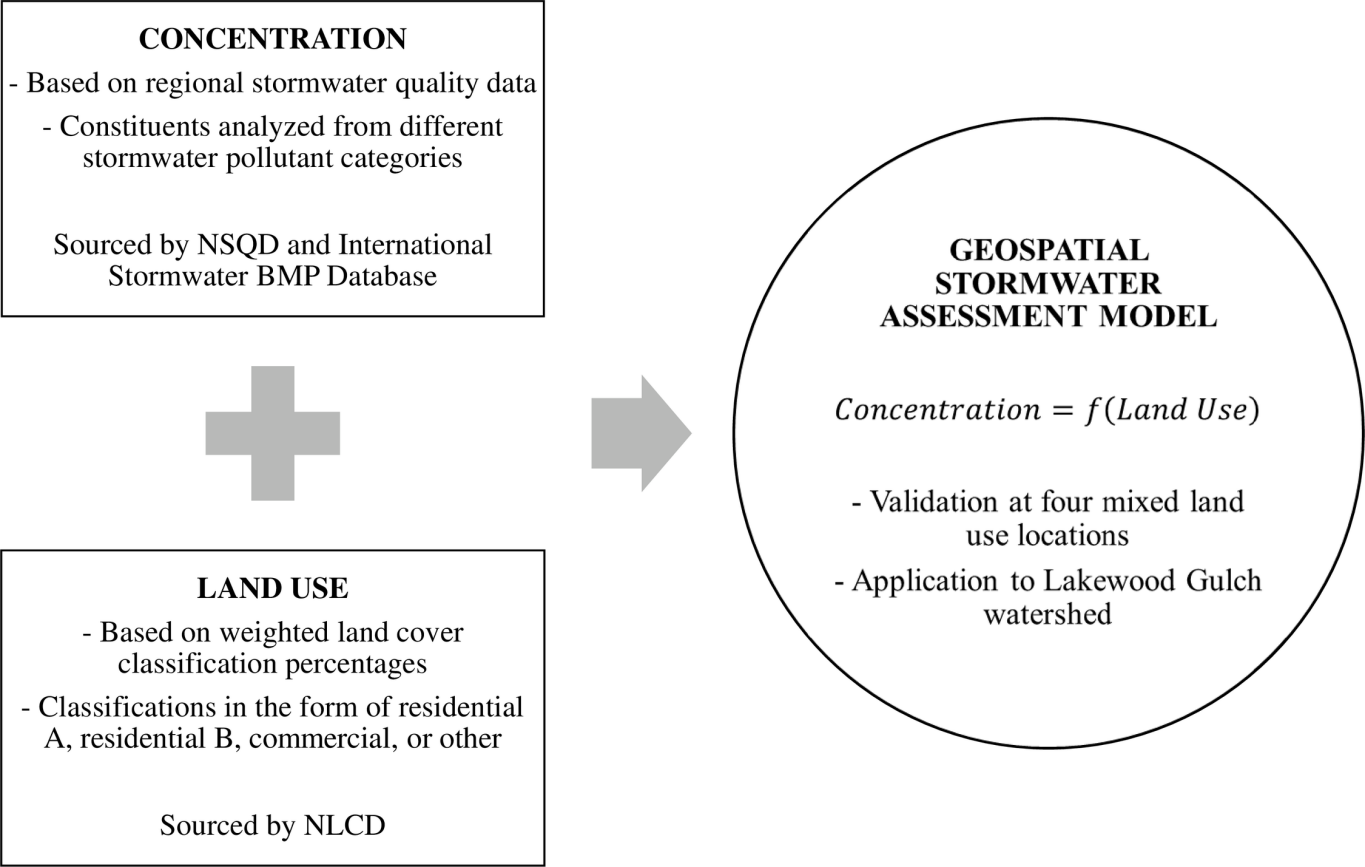
Conceptual model for nonpoint stormwater concentration with land use.

This section presents a three-phase process to predict nonpoint stormwater runoff quality from land use (Fig 1). The first phase is a nonpoint stormwater quality analysis to query, locate and evaluate regional stormwater samples for residential and commercial land uses within an area of interest. The second phase is a geospatial land cover analysis of data sets collected and analyzed using geographic information systems. The third phase is to perform regression of the concentration data on land use data. This regression then provides an estimate of stormwater runoff quality for each constituent of interest. The following subsections elaborate each of these three phases.

### 2.1 Nonpoint stormwater quality analysis

Stormwater runoff quality data in the United States are largely available in at least one of two Federally-sponsored databases. First, the National Stormwater Quality Database (NSQD) is a national urban stormwater runoff database that serves as an important resource for urban runoff data, categorized by location, land use, years of record, and several other characterizations [37,38]. Recently updated to Version 4.02, the NSQD is the major resource for all stormwater data collected over recent years. Second, the International Best Management Practices Database provides stormwater monitoring data collected at monitored BMP sites [39]. These sites, more often than not, have a reference site to which the BMP system is compared. This reference site, where stormwater quality samples are also taken, is used as a resource within the NSQD. Both databases are primary resources used in this study as they both address urban runoff pollutant concentration as a function of land use, where pollutants are grouped into categories associated with stormwater runoff, including sediments, nutrients, and metals [10,37,38].

Selection of relevant stormwater sampling locations is required prior to analyzing runoff concentration data. Stormwater sampling locations are selected using the following four criteria: (1) site location within the area of interest, (2) data available for the three pollutant categories of interest—sediments, nutrients, and metals—, (3) at least two years of data, and (4) land use indicated in the record. The last of these criteria, regarding land use, provides the basis for the correlation model (Fig 1). Selection of stormwater sampling locations according to these four criteria is possible using a query-based selection process using the NSQD v4.02. Having identified sampling locations, concentration data for the constituents of interest can be extracted. A quality assurance check is required to identify repeated or missing values and variations in monitoring practices between the two databases.

**Fig 2:**
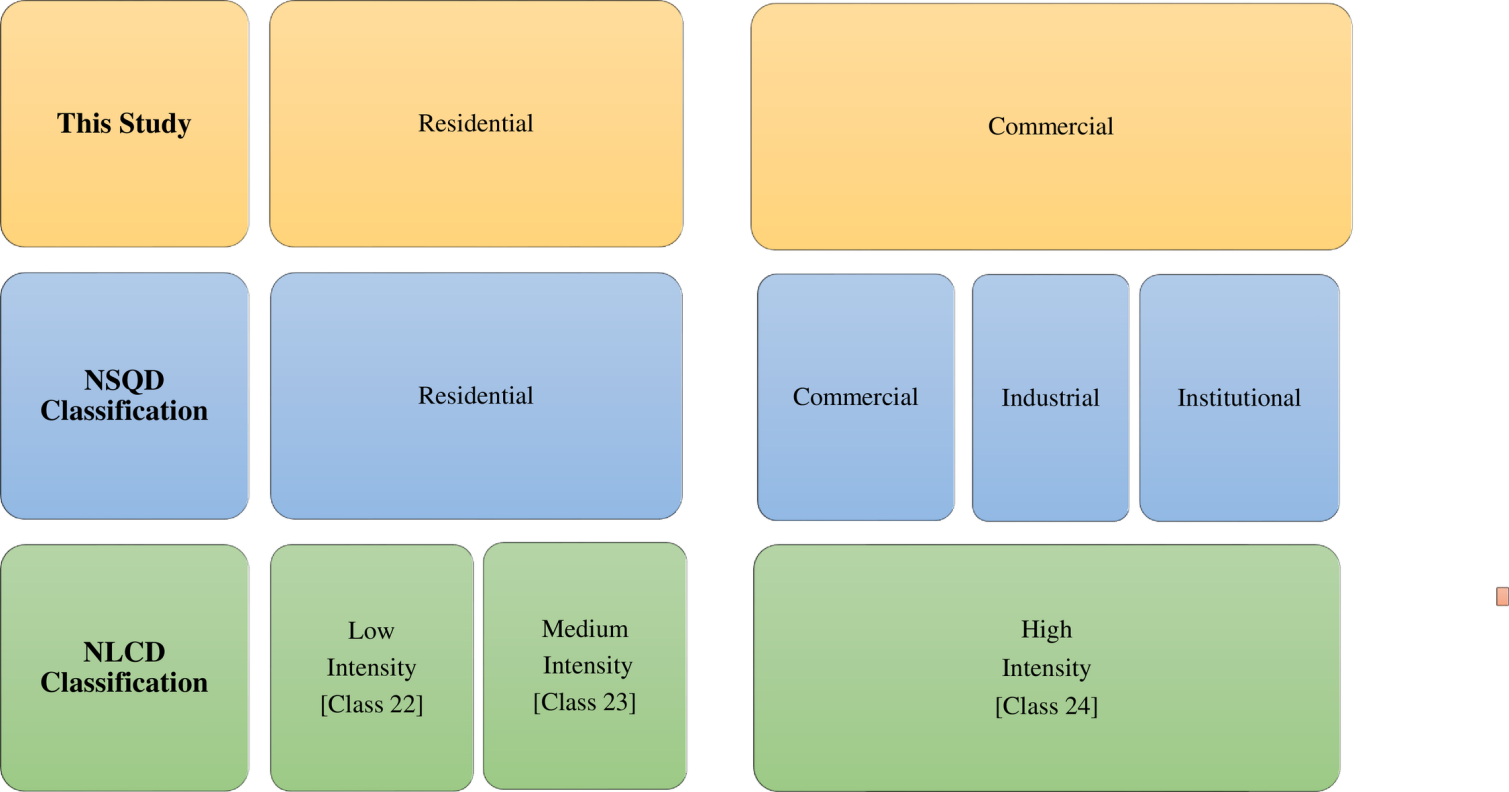
Breakdown of classifications and links between NLCD and NSQD.

To correspond with land use categories used in the NLCD, the NSQD categories of commercial, industrial, and institutional were combined into a single category with high imperviousness (≥80%) and labeled commercial (Fig 2). To account for a mixed land use, a combination of commercial, residential, and other class type cells would need to be expressed as a vector of percentages corresponding to each land use and summing to 100%. In the current application, only non-mixed NSQD land use categories were used, meaning statistical analysis and application from sample sites were 100% residential, 100% commercial, or 100% open space.

To illustrate these methods, consider an example application to metropolitan Denver, Colorado. Using the NSQD v4.02 as the main stormwater database, stormwater sampling locations were selected through the following queries from the 690 sites in the data base to 13 sites regionally similar to an application example for Denver, Colorado. The query process was first by county (Adams, Arapahoe, Denver, or Jefferson), then by complete land use type (100% commercial, 100% residential, or 100% open space) with at least two years of sampling data. There were two locations, Urban Drainage and Flood Control District (UDFCD) Modular Porous Pavement and Shop Creek Wetland Pond, with two data sets reported for different sampling years. Since these two sites were sampled over different monitoring periods at the same locations, datasets for each were combined into a single dataset for each location. This reduced the site locations from 13 to 11 with the UDFCD Modular Porous Pavement and Shop Creek Wetland Pond sites having each with one dataset summary for two monitoring periods. Overall, the selection identified 11 sampling locations that consist of five 100% residential sites, five 100% commercial sites and one 100% open space site represented in Table 1.

**Table 1:** Overview of residential and commercial stormwater sampling locations.

Site ID^1^^,^^2^	Site Name	Years of Record	Location	No. of Events
***Residential***				
**COLAIRIS**	21^st^ and Iris Rain Garden	2011–2014	39.7488 N 105.1066 W	54
**CODEGRHE**	Grant Heron	2000–2009	39.6197 N 105.0582 W	29
**CODEGRRE**	Grant Reflect	2000–2009	39.6184 N 105.0594 W	25
**COAUSHCR**	Shop Creek Wetland Pond^3^	1990–1997	39.6291 N 104.7415 W	55
**CODEORPO**	UDFCD Orchard Pond	2000–2011	39.6211 N 105.0598 W	106
***Commercial***				
**COACWWL3**	Arapahoe Country Water & Wastewater Authority (L3)	2008–2009	39.6005 N 104.8379 W	19
**COACW6W7**	Arapahoe Country Water & Wastewater Authority (W6W7)	2008–2009	39.5919 N 104.8206 W	17
**CODEWAWA**	Denver Wastewater Building	2008–2014	39.7209 N 105.0106 W	67
**COLASHOP**	Lakewood Shops	2005–2014	39.8748 N 105.1630 W	121
**COLAMOPA**	UDFCD Modular Porous Pavement^3^	1994–2006	39.8833 N 105.2000 W	48
***Open Space*^4^**				
**COFEMORR**	Rooney Gulch at Rooney Ranch near Morrison	1980–1981	39.6908 N 104.1922 W	7

^1^ Sampling data and site locations from [38,39,40]. Size of storm events can vary based on data reported to the NSQD. In general, sites are commonly set up to capture events greater than the water quality event.

^2^ Site ID taken directly from NSQD v4.02 or if newly added site, similar site ID was created.

^3^ Two sites, Shop Creek Wetland Pond and UDFCD Modular Porous Pavement, had two sets of sampling years listed in the NSQD v4.02, however, the non-continuous records have been reported together as one sampling location for each site by land use type within this study.

^*4*^ Open space site data were added to this table for mixed land use validation. Statistical analysis has previously been reported for the regional site–COFEMORR: Rooney Gulch at Rooney Ranch near Morrison–in past reports [40].

Within the three pollutant categories of sediments, nutrients, and metals, seven stormwater constituents of interest were identified (Table 2). The NSQD v4.02 had not been updated with sampling events from 2014, specifically for UDFCD stormwater monitoring sites. Thus, raw data from the 2014 sampling season was added manually as well as backfilling missing entries not recorded (personal communication, H. Piza, Urban Drainage and Flood Control District, Denver, Colorado and J. Clary, Wright Water Engineers, Denver, Colorado). All sampling data for the seven stormwater pollutants is provided in S1 and S2 Tables, which correspond to the sites’ primary data source location and final data used for the stormwater quality analysis, respectively. With all stormwater quality analysis criteria addressed, final data sets on all sampling events for each of the eleven sites was complete.

**Table 2:** Common urban stormwater pollutants and potential sources.

Type	Symbol	Pollutant	Potential Source ^1^
**Sediments**	TSS	Total Suspended Solids	Construction sites, erosion, poorly vegetated lands, large commercial vehicles
**Nutrients**	TKN	Total Kjeldahl Nitrogen	Lawn fertilizers, domestic animal waste, vegetative matter, detergents
	NO_2_+NO_3_	Nitrite + Nitrate	
	TP	Total Phosphorus	
	DP	Dissolved Phosphorus	
**Metals**	Cu	Total Copper	Industrial processes, vehicles, soil erosion, and atmospheric deposition from fuel combustion.
	Zn	Total Zinc	

^1^ Data for potential sources of common stormwater pollutants compiled from [10,17,25,29,41].

### 2.2 Geospatial land cover analysis

Supported by the U.S. Department of the Interior and the U.S. Geological Survey, the Multi-Resolution Land Characteristics Consortium specifies land covers for uses ranging from agricultural to forest to developed areas in their National Land Cover Database (NLCD). Land use data were taken from the NLCD, which provides 30 m by 30 m gridded cell resolution for each of the land cover classifications. To limit the quantity of data requiring analysis, only developed land uses were analyzed as they are the key locations for urbanization. These land uses, which include the standard NLCD classifications developed low intensity, developed medium intensity, and developed high intensity, were used to correlate with the land use runoff data from the stormwater quality analysis. To allow correlation across the different land use classifications in the NLCD and the NSQD, the NLCD classifications “developed low intensity” and “developed medium intensity” correspond to the NSQD residential classification, while the NLCD classification “developed high intensity” corresponds to the lumped commercial classification (Fig 2).

The last process in the geospatial analysis used the union tool in ESRI ArcMap 10.3 to join overlapping features into a new output feature class, from which final land use areas and percentages could be calculated for the different sub-basins. These sub-basins land use areas and percentages will be used for the geospatial stormwater assessment method further explained in the next section.

**Fig 3:**
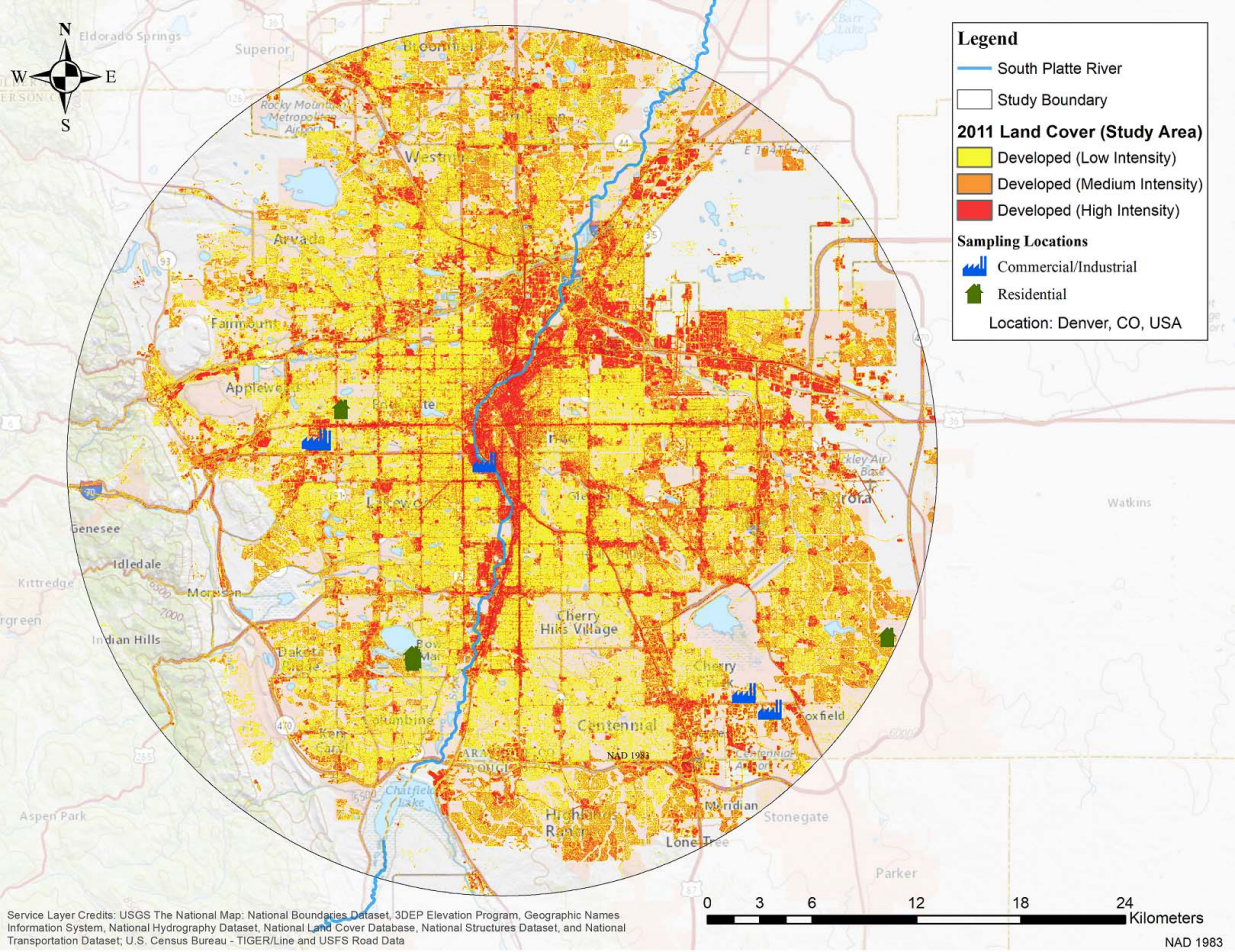
Sampling locations and land use classes within 25 km of Denver, Colorado. Used with permission. Copyright 2018 ESRI, ArcGIS Online, USGS, National Boundaries Dataset, 3DEP Elevation Program, Geographic Names Information System, National Hydrography Dataset, National Land Cover Database, National Structures Dataset, and National Transportation Dataset, U.S. Census Bureau—TIGER/Line and USFS Road Data and the GIS User Community.

To illustrate these methods, consider once again metropolitan Denver, Colorado. Using the NLCD, a clipped raster was created for the area within a 25 km radius of the Colorado capitol building on Lincoln Street between 14^th^ Avenue and Colfax Avenue (Fig 3), which returned over 180,000 polygons for developed land use classifications. Reducing the number of polygons to a more manageable number without reducing cell raster sizes, a watershed within the 25 km study boundary was selected to apply this concept and analysis.

**Fig 4:**
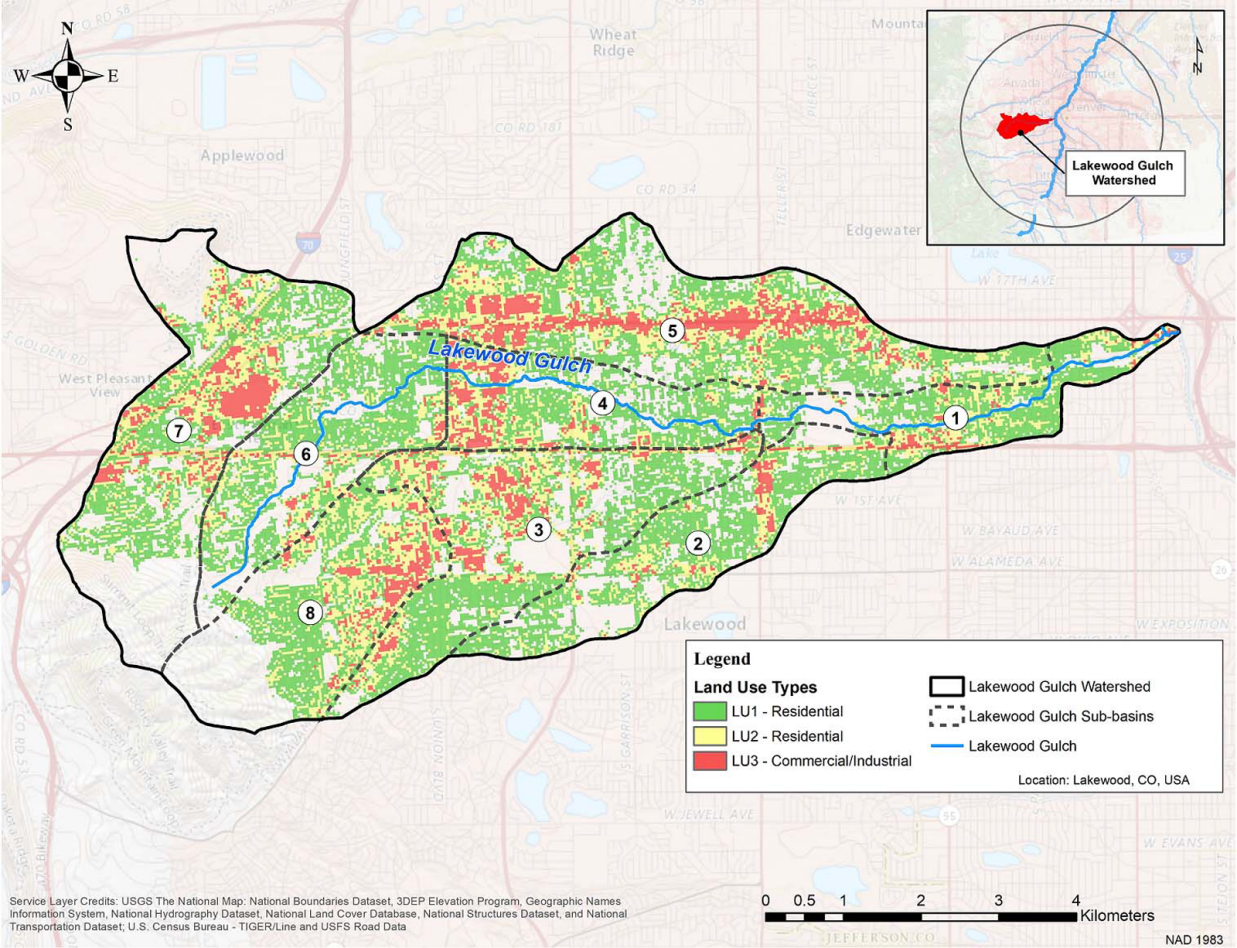
Map of developed Lakewood Gulch watershed with sub-basins and land use. Used with permission. Copyright 2018 ESRI, ArcGIS Online, USGS, National Boundaries Dataset, 3DEP Elevation Program, Geographic Names Information System, National Hydrography Dataset, National Land Cover Database, National Structures Dataset, and National Transportation Dataset, U.S. Census Bureau—TIGER/Line and USFS Road Data and the GIS User Community.

The Lakewood Gulch watershed is situated west of Denver and is for the most part a mixed residential and commercial area. The total area of the watershed is 51 km^2^ with a main channel length of 16 km (Fig 4). After removing unwanted classifications from the 2011 NLCD raster dataset, the raster to polygon tool was used in ArcMap 10.3 to convert all cells classified as developed into 4,000+ polygons. An attribute table was then created for these polygons that could be exported into a spreadsheet to identify developed land use areas and percentages for the watershed. To join overlapping features into a new output feature class, ArcMap 10.3’s union tool used two inputs to address overlapping layers, (1) the polygon features created in the previous step for the Lakewood Gulch watershed, and (2) a polygon shapefile of eight delineated sub-basins within the watershed.

### 2.3 Regression of concentration data on land use data

To recapitulate the three-phase process illustrated in Fig 1, the first phase identified nonpoint stormwater runoff concentrations, *C*. In parallel, the second phase identified the fractional distribution of land use, *LU*_i_, where the subscript *i* denotes the land use. These elements come together in the third phase, which fits correlation coefficients *X*_i_ to predict nonpoint stormwater runoff concentrations for each constituent from land use, where again the subscript *i* denotes the land use. The general correlation equation can be written as

$$C=\sum LU_{i}X_{i},$$ (1)

where the concentration *C* is the dependent variable, the fractional distribution of land use *LU*_i_ is the independent variable, the correlation coefficients *X*_i_ are fitted by least squares, and the intercept is zero by construction.

In the example application, there are three different land uses. Using these land uses, where *i* = 1 is residential, *i* = 2 is commercial, and *i* = 3 is open space, the correlation equation becomes

$$C=LU_{1}X_{1}+LU_{2}X_{2}+LU_{3}X_{3},$$ (2)

which can be written in matrix notation as

[C]=[LU][X] (3)

where *X* is a 3×1 vector. If *C* is another 3×1 vector and *LU* is a 3×3 matrix representing a system of three independent linear equations, then the correlation coefficient vector *X* will have a unique solution. In the more general case where there are multiple concentration measurements for each catchment (*i*.*e*., for each combination of land uses) then the system will be overdetermined, in which case *X* is fitted by least squares. For example, consider an area with three land uses and two catchments. In this case, for each constituent, the matrix Eq (3) could be expanded as

$$\left[\begin{array}{c} C_{1,1}\\ C_{1,1}\\ \begin{array}{c} \begin{array}{c} C_{1,1}\\ \vdots \\ C_{2,1} \end{array}\\ C_{2,1}\\ C_{2,1} \end{array} \end{array}\right]=\left[\begin{array}{c} \begin{array}{ccc} LU_{1,1} & LU_{2,1} & LU_{3,1} \end{array}\\ \begin{array}{ccc} LU_{1,1} & LU_{2,1} & LU_{3,1} \end{array}\\ \begin{array}{c} \begin{array}{c} \begin{array}{ccc} LU_{1,1} & LU_{2,1} & LU_{3,1} \end{array}\\ \vdots \\ \begin{array}{ccc} LU_{2,1} & LU_{2,2} & LU_{2,3} \end{array} \end{array}\\ \begin{array}{ccc} LU_{2,1} & LU_{2,2} & LU_{2,3} \end{array}\\ \begin{array}{ccc} LU_{2,1} & LU_{2,2} & LU_{2,3} \end{array} \end{array} \end{array}\right]\left[\begin{array}{c} X_{1}\\ X_{2}\\ X_{3} \end{array}\right]$$ (4)

where *C*_1,1_ is from catchment 1 sample 1, *C*_1,2_ is from catchment 1 sample 2, and so on; and *LU*_1,1_ is from catchment 1 land use 1, *LU*_1,2_ is from catchment 1 land use 2, and so on. Note all samples from a given catchment have identical land use distributions that sum to unity across each row (*e*.*g*., *LU*_1,1_ + *LU*_1,2_ + *LU*_1,3_ = 1). The correlation coefficient *X* can be fitted in a least squares sense using MATLAB’s function FITLM [42].

### 2.4 Model validation

Validation of the regression required returning to the NSQD to locate mixed use sampling locations from which predicted and measured values could be compared on the basis of solute mass transported in a given storm event:

$$M=C*V_{runoff}$$ 5

where *M* represents the total nonpoint mass of loading, *C* represents total nonpoint loading concentrations (weighted from matrix) and *V*_runoff_ represents volume of runoff for the storm event, taken from observed rainfall over the study area (reported for each event in NSQD).

**Fig 5:**
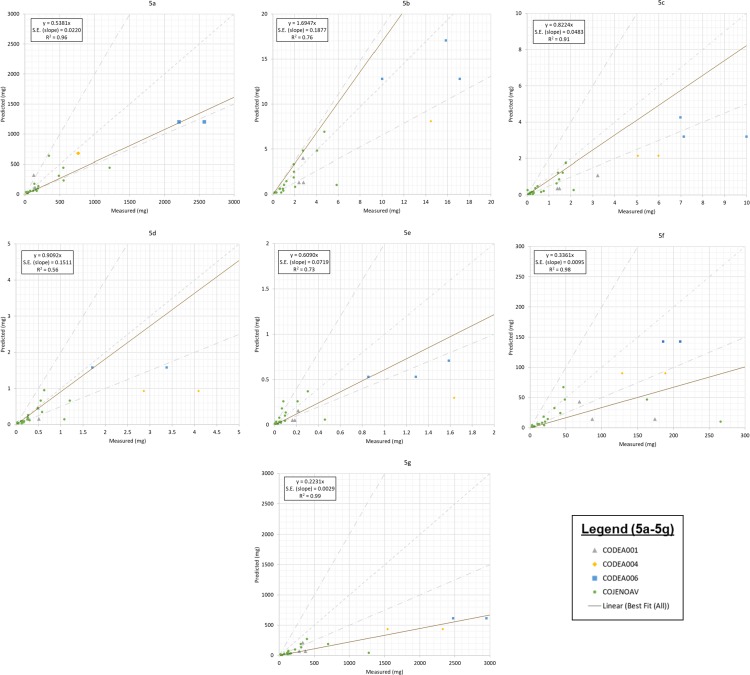
Predicted versus observed loading discharged at the validation sites. The dashed line is a 1:1 slope for equal predictions and observations. The skipped dashed lines are 2:1 and 1:2 slopes that delineate the region in which the model is accurate within a factor of two. The dotted line is a linear regression, including data not plotted on the axes shown. Plots are represented as (a) TSS; (b) TKN; (c) NO2+NO3; (d) TP; (e) DP; (f) Total Copper, and (g) Total Zinc. With the axes chosen, twelve data points do not appear: (14896, 8,669) and (5993, 1603) for TSS; (40.3, 103.41) and (21.4.2, 8.1) for TKN; (28.2, 27.4) for NO2+NO3; (5.40, 2.10) for TP; (2.67, 0.30) and (4.03, 3.80) for DP; (3,462.4, 1,153.3) and (419.0, 189.8) for Cu; and (24,961.7, 5,576.7) and (3,745.5, 816.8) for Zn.

For validation, new sampling locations were identified following a modified version of the site selection process, which was altered by removing two previous selection constraints–the constraint of requiring non-mixed land use and the constraint of requiring at least two years of data. Using these modified selection criteria, the final four mixed land use stormwater sampling locations for validation included: (1) Cherry Creek Storm Drain at Colfax Avenue, (2) Sand Creek Tributary at 34^th^ and Havana, (3) South Platte River Storm Drain at 54^th^ and Steele, and (4) North Avenue Storm Drain at Denver Federal Center (Table 3). There was a total of twenty-nine (29) sampling events collected between the four sites with twenty (20) collected at the Denver Federal Center. All stormwater quality data and storm event runoff is reported in the supporting information (S3 Table). The relationship between predicted and measured values is shown in Fig 5 for each of the constituents. Goodness-of-fit is quantified using a scaling factor, *k*, such that,

$$k=M_{predicted}/M_{measured}$$ (6)

where *M*_predicted_ represented the stormwater matrix predicted mass loading and *M*_measured_ represents the actual mixed land use mass loading. Using this framework, *k* < 1 corresponds to underprediction, *k* > 1 corresponds to overprediction, and ½ < *k* < 2 corresponds to predictions within a factor of two. Fitted values of *k* corresponding to the results are shown in boxes within Fig 5, in addition to correlations and standard errors of the regression slope.

**Table 3:** Summary of mixed land use validation sites from NSQD v4.02.

Site ID	Site Name	Drainage Area (km^2^)	Percent Residential (%)	Percent Commercial (%)	Percent Open Space (%)
**CODEA001**	Cherry Creek Storm Drain at Colfax Ave	0.7	5	87	8
**CODEA004**	Sand Creek Tributary at 34th and Havana	2.5	0	89	11
**CODEA006**	South Platte River Storm Drain at 54th and Steele	3.1	6	65	29
**COJENOAV**	North Avenue Storm Drain at Denver Federal Center	0.3	33	30	37

### 2.5 Statistical analysis

Statistical analysis was performed separately for the three different land use types–residential, commercial and open space–to determine event mean concentrations and event median concentrations. Both event mean and median concentrations are provided in the stormwater quality analysis as the process and framework is solely based on the user’s preference and the dataset available to provide accurate assessment inputs. In other words, an appropriateness check for the data should be considered based on sampling events (i.e. event median preferred over event mean in cases where water quality data has high skewness). P-values to determine the significance of the difference between mean concentrations for residential and commercial land uses were calculated with a one-sided *t*-test (S4 Table).

## 3. Results

### 3.1 Nonpoint stormwater quality results

Concentrations from nonpoint residential areas were greater than commercial areas for TKN, NO_2_+NO_3_, TP, and DP; concentrations were not significantly different for TSS, Cu, and Zn; both at *α* = 0.05 level. Commercial runoff concentrations were not significantly higher than residential for any of the seven constituents analyzed in this particular example. In addition, linear regression was used to check for significant correlations between (1) TKN and NO_2_+NO_3_, (2) TP and DP, and (3) Zn and Cu. Such correlations, if significant, allow simplified modeling, for example, using the co-pollutant feature in EPA’s Storm Water Management Model to determine relations for the constituent sets [1]. Regression statistics, including the standard error of the slope, were calculated (S5 Table). Correlation slopes were positive and significant at the *α* = 0.05 level for TP versus DP and for Zn versus Cu, but not for TKN versus NO_2_+NO_3_.

### 3.2 Geospatial land cover results

Using data from the 2011 NLCD, this study area with a radius of 25 km is 43.2% residential, including 26.6% with NLCD classification “developed low intensity” and 16.6% “developed medium intensity’ (Fig 3). The study area, with total area of 1,963 km^2^, is 6.3% commercial from the NLCD classification “developed high-intensity”. The remaining 50.6% is classified as land cover with less than 20% imperviousness.

### 3.3 Model application to smaller watershed

As stated, a watershed was selected for model application. The Lakewood Gulch watershed is a tributary of the South Platte River. The watershed is located just west of central Denver (Fig 4), is classified by the U.S. EPA’s *My WATER Mapper* as impaired in the category of Aquatic Life Warm Water and in the category of Recreational Primary Contact. Eight sub-basins, which have areas ranging from 3.5 km^2^ to 9.7 km^2^, were delineated with the geographic information systems software add-in ArcHydro [43]; ArcHydro was not used in the subsequent analysis of recommended BMP locations. Sub-basins are shown on Fig 4, and their statistics are summarized in supporting information (S6 Table). The normalized sub-basins of Lakewood Gulch were compared to one another to geographically evaluate and identify key contributing areas within the watershed that could potentially be key contributors to unregulated, nonpoint urban stormwater impairments.

**Fig 6:**
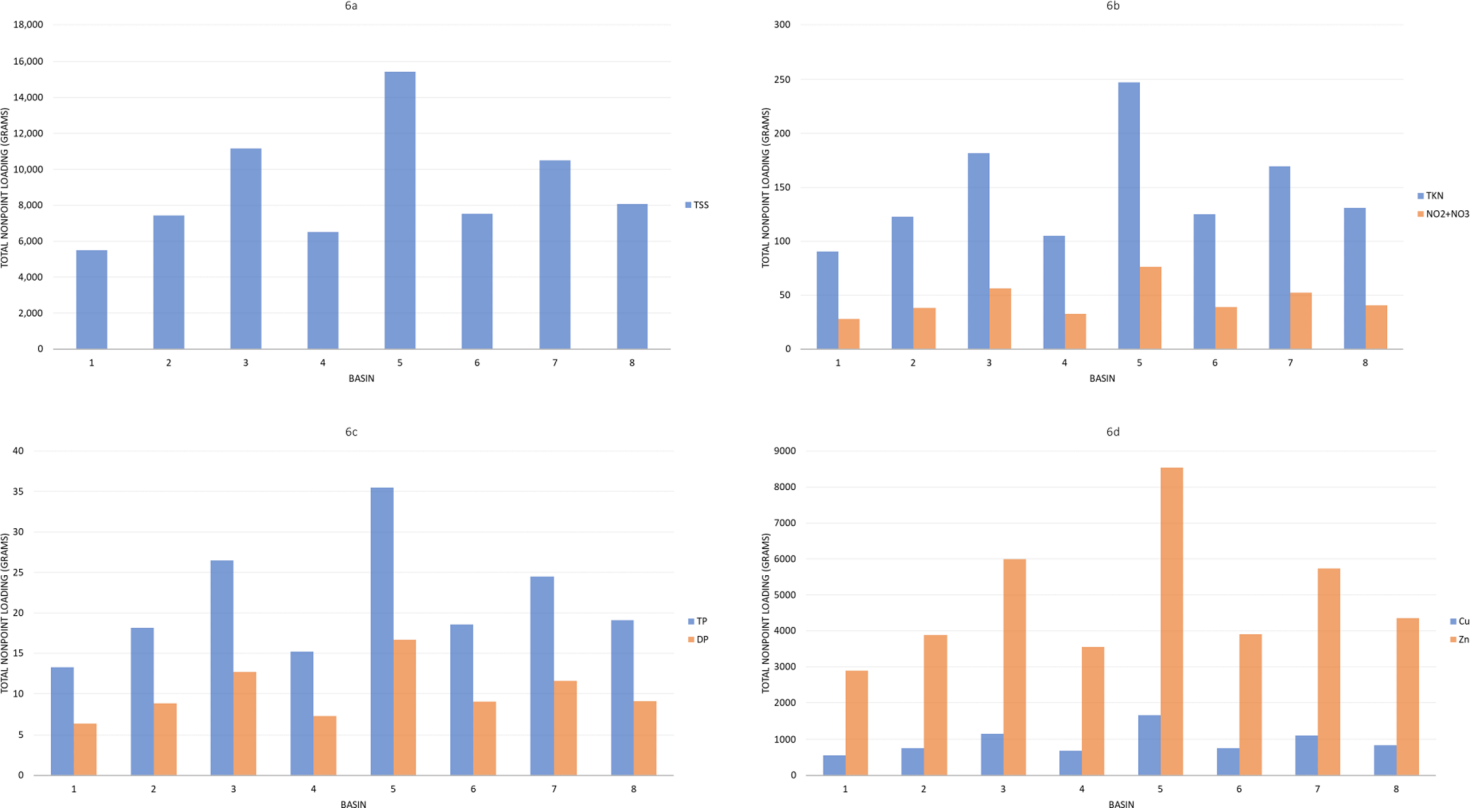
Application of the nonpoint stormwater quality assessment matrix to Lakewood Gulch. The four different graphs represent loading for the seven constituents within each sub-basin shown in Fig 5. Bar charts are shown for (a) TSS, (b) TKN and NO2+NO3, (c) TP and DP, and (d) Total Zinc and Total Copper. For this example, event mean concentrations were used with a 1-cm runoff storm event.

Nonpoint stormwater runoff concentration was predicted for each of the seven constituents in Table 2 and each of the eight sub-basins in Fig 4 using the land use percentages from the NLCD and the coefficient vector *X* calibrated over the total 55 km^2^ study area for Lakewood Gulch. From this analysis, Sub-Basin 3, Sub-Basin 5, and Sub-Basin 7 are the key locations within the watershed with priority for BMP implementation with respect to developed residential and commercial land use for the various constituent distributions (Fig 6). Sub-Basin 5, which has the largest area (both for residential and commercial land use), would provide the highest pollutant loadings from nonpoint runoff using this preliminary assessment approach. The prioritization of these three sub-basins allows for a general focus area for additional spatial analyses to be performed with objectives to locate micro-geographical regions contributing the greatest pollutant loading within these sub-basins.

## 4. Discussion

Research on unknown sources of contamination continues to drive the U.S. EPA to set stricter regulations as amendments to the Clean Water Act look to restore natural streams to their pristine state. Although efforts on reviving damaged or lifeless ecosystems remain costly and time extensive, growing surface water impairments continue to drive urban planners, environmentalists, and water resource engineers to look for new methods to treat urban impacts from decades of urban pollution. In this context, the method presented here may prove useful as a straightforward initial analysis that can be applied regionally without requiring the collection of compilations of additional water quality and land use measurements. This method is not intended to calculate exact values reaching streams from nonpoint sources, but rather to provide a base representation and standardization of a given area to determine how each sub-area contributes in reference to another.

The parsimonious model presented in this manuscript relies on the essential principle that nonpoint stormwater runoff concentrations can be predicted from land use types and appropriate actions can be made to aid the preservation and restoration of the urban hydrologic regime. From this, the community can create a well-documented urban regional model to guide implementation of different BMPs to reduce pollutants reaching surface waters allocating funds to areas contributing higher percentages of stormwater pollutants in the watershed. This model does not account for any engineered stormwater quality treatment such as water quality detention basins, rain gardens or other water quality treatment features. However, it does enable users, when analyzing at a sub-basin level, to identify high contributing areas to surface waters and custom tailor the base function of the model to their evaluation needs.

Uncertainty should be addressed when applying this model to large, poorly documented regions from which approximated values might differ substantially what actually occurs on the surface. For example, in the case of the Lakewood Gulch watershed discussed above, if additional data were available for the non-residential, non-commercial areas shown in grey on Fig 4, a complete nonpoint watershed loading model could be developed reducing uncertainty between results. One should also bear in mind that this approach assumes that at least one solute concentration differs by land use. Additionally, the validation of regionally appropriate land use data is constrained to only one sampling location for open space areas that met requirements for water quality sampling data. Future assessments should expand boundary limits with limited data sets and/or incorporate newly collected data into the study for a more regionally appropriate value for these cases. Alternatively, the national average can always be used.

The methodology is not intended to single-handedly predict runoff concentrations with any degree of precision. Instead, the methodology can be used to help initial identification and standardization of areas of concern. Additionally, the universal function of the approach allows for scalability between all models and scales in a watershed. Coupling this initial approach with additional analysis of receiving streams would provide significant additional benefit, as only in-stream stormwater sampling can verify results predicted by the methodology presented here. To elaborate, tracing pollutants to several areas in a watershed using a post-storm mass balance approach (i.e. major outfalls, tributaries, etc.) can provide physical water quality sampling that is representative of various contributing areas and then results could be calibrated for this model. In this context, the availability of a low-cost initial identification of BMP locations would be immensely valuable, particularly considering that installation and maintenance of stormwater BMPs can greatly vary based on the type of BMP that is selected.

From the application to the Lakewood Gulch watershed, the assessment identifies Subbasin 3 and Subbasin 5 contributing high sediment and metal pollutant loads to the watershed during a storm event. Subbasin 3 is a highly impervious and excessively traveled area comprising a main roadway into Denver as well as some residential area. Subbasin 5 is also highly impervious and excessively traveled, comprising commercial outlets and high commercial areas. Further analysis of each of these subbasins, which would ideally overlay known monitoring locations, storm drainage networks, stormwater problems, and topographic assessments for flow paths, will then identify the known inlets and outlets collecting and contributing the high pollutant loads in these watersheds and inform what BMP can be deployed to implement the philosophy of green infrastructure. For Subbasin 3, BMPs targeting metals and sediments in space-constrained areas would be recommended (i.e. microsystems for inlets and sediment collection). For Subbasin 5, less space-constrained areas are required, resulting in a similar BMP type that can serve a larger drainage area with effective water quality treatment (i.e. detention, retention, and wetland ponds).

With proper planning, evaluation and selection of BMP designs and strategies, installation and retrofits of efficient stormwater treatment systems can play key roles as populations and developments continue to grow and encroach on natural ecosystems. Learning to understand impacts prior to BMP installation can be a key factor for evaluating stormwater runoff and also to provide treatment during minor events. As such, the method presented here supports the broader philosophy of contemporary urban hydrology, to protect and restore principles of the natural hydrologic regime.

## 5. Conclusions

The methodology presented here comprises three phases (Fig 1). In the first phase, regional stormwater quality analysis provides summary statistics, identification of significant differences between developed land uses, and linear regression to identify co-pollutants. In the second phase, land cover classifications are used to determine land use percentages for a given study area. Once all data are collected, the third phase uses Eq (4) to predict nonpoint stormwater runoff quality from land use.

This straightforward model uses land use percentages as weighting coefficients to evaluate urban watersheds for potential pollution from nonpoint stormwater runoff. This method can be used to identify watersheds or smaller sub-basins that are major contributors of pollutants from unregulated nonpoint locations in urban areas with multiple constituents and multiple land uses. With the appropriate data, the model can be applied beyond planning and preliminary assessments and scaled down to subbasin and block-level assessments where known stormwater problems exist, such as flooding problems from directly connected impervious areas, mass balance in combined sewer overflows, or areas draining to an inlet. Additionally, if this method can be coupled with a topographic assessment, understanding site-level flow paths can further influence the green infrastructure applications that strive to reduce runoff as close to the source as possible. As models, research and data, and methods improve, implementation of water quality treatment features and integrated prevention plans will continue to expand as protection of natural lands and restoration of the urban regime will continue to remain a key objective for planners, environmental scientists, and engineers who develop design and standards for sustainable urban infrastructure.

## Supporting information

S1 Table Overlap and location summary for stormwater quality analysis. (PDF)

S2 Table Raw sampling data from NSQD, IBMP, and UDFCD. (PDF)

S3 Table Raw sampling data from NSQD for validation using mixed land use sites. (PDF)

S4 Table Statistical analysis for all constituents. (PDF)

S5 Table Summary of linear correlations and relationships between similar constituents in developed land use classes. (PDF)

S6 Table Overview of geospatial analysis for application to Lakewood Gulch watershed. (PDF)

S1 File ESRI copyright permission letter. This file includes a copyright permission letter from Environmental Systems Research Institute (ESRI) allowing permission to publish the basemap used with Figs 3 and 4.
(PDF)

S2 File MATLAB code and supporting data files. The zip folder includes a read-me text file, the MATLAB code (as .m file), fourteen (14) supporting data files (as .txt for each constituent concentration and land use percentage), and a published MATLAB document (as .pdf) representing MATLAB’s run of the code using supporting data files and output of results from statistical analysis.
(ZIP)
