# Supplementary material for: Predicting nonpoint stormwater runoff quality from land use
Source: PLoS One. 2018 May 9;13(5):e0196782. doi: 10.1371/journal.pone.0196782 (PMC5942771; doi:10.1371/journal.pone.0196782)
Supplement: S1 Table — (PDF) [file pone.0196782.s001.pdf]

S1 Table. Overlap and Location Summary for Stormwater Quality Analysis

Site Locations	NSQD & IBMP	UDFCD & WWE et al. 2013
21st and Iris Rain Garden	x	x
Grant Heron	x	
Grant Reflect	x	
Shop Creek Wetland Pond	x	
UDFCD Orchard Pond	x	
Arapahoe County Water & Wastewater Authority (L3)	x	
Arapahoe County Water & Wastewater Authority (W6W7)	x	
Denver Wastewater Building	x	x
Lakewood Shops	x	x
UDFCD Modular Porous Pavement	x	
Rooney Gulch near Rooney Ranch	x	x
