# Supplementary material for: Predicting nonpoint stormwater runoff quality from land use
Source: PLoS One. 2018 May 9;13(5):e0196782. doi: 10.1371/journal.pone.0196782 (PMC5942771; doi:10.1371/journal.pone.0196782)
Supplement: S2 Table — (PDF) [file pone.0196782.s002.pdf]

S2 Table. Raw Sampling Data from NSQD, IBMP, and UDFCD

Site ID	Land Use	Date of Sample	TSS (mg/L)	TKN (mg/L)	NO_2+NO_3 (mg/L)	TP (mg/L)	DP (mg/L)	Tot. Cu (µg/L)	Tot. Zinc (µg/L)
COLAIRIS	RE	07/27/13	205	3.20	0.03	0.36	0.01	9	69
COLAIRIS	RE	08/03/13	790	3.80	0.03	0.78	0.07	23	173
COLAIRIS	RE	08/11/13	568	4.80	0.04	0.75	0.02	33	252
COLAIRIS	RE	05/31/14	518	3.10	0.11	0.52	0.08	18	116
COLAIRIS	RE	09/11/12	179	0.90	0.23	-	0.14	7	-
COLAIRIS	RE	07/13/13	641	3.50	0.33	0.44	-	15	91
COLAIRIS	RE	07/31/12	161	2.80	0.36	-	0.46	14	-
COLAIRIS	RE	05/19/11	64	1.10	0.37	0.20	0.08	8	44
COLAIRIS	RE	05/20/13	1310	13.40	0.40	1.91	0.05	46	308
COLAIRIS	RE	07/13/11	476	2.40	0.41	0.55	0.09	23	123
COLAIRIS	RE	09/22/13	19	1.40	0.44	0.18	0.14	5	23
COLAIRIS	RE	06/04/13	149	1.80	0.44	0.27	0.10	11	56
COLAIRIS	RE	07/16/14	430	5.00	0.47	0.72	0.07	30	239
COLAIRIS	RE	05/12/12	21	1.10	0.49	-	0.16	5	-
COLAIRIS	RE	05/11/12	40	1.10	0.50	0.15	0.09	6	-
COLAIRIS	RE	05/11/11	70	1.50	0.50	0.25	0.16	30	49
COLAIRIS	RE	05/09/14	415	3.20	0.51	0.80	0.13	28	190
COLAIRIS	RE	07/12/11	947	3.00	0.55	0.71	0.07	31	166
COLAIRIS	RE	07/30/14	84	1.80	0.56	0.27	0.13	10	71
COLAIRIS	RE	06/16/13	166	6.80	0.56	0.85	0.37	31	112
COLAIRIS	RE	05/06/12	127	2.40	0.57	0.39	0.22	12	68
COLAIRIS	RE	09/10/14	158	1.30	0.58	0.21	0.16	7	37
COLAIRIS	RE	09/05/14	48	1.60	0.59	0.22	0.12	14	70
COLAIRIS	RE	05/18/11	232	1.80	0.59	0.38	0.12	17	94
COLAIRIS	RE	07/17/14	193	2.50	0.63	0.30	0.07	17	96
COLAIRIS	RE	06/19/11	274	4.50	0.63	0.75	0.33	20	148
COLAIRIS	RE	07/07/11	766	1.80	0.65	0.39	-	11	-
COLAIRIS	RE	09/09/13	52	1.10	0.67	0.16	0.07	5	21
COLAIRIS	RE	05/09/13	318	3.00	0.68	0.49	0.09	22	137
COLAIRIS	RE	06/09/14	265	3.70	0.72	0.61	-	20	146
COLAIRIS	RE	05/05/12	641	5.80	0.73	1.00	0.22	28	191
COLAIRIS	RE	07/26/11	158	3.10	0.76	0.36	0.05	17	102
COLAIRIS	RE	08/11/12	108	3.40	0.76	-	0.38	9	59
COLAIRIS	RE	06/19/14	261	5.10	0.77	0.65	0.18	19	159
COLAIRIS	RE	05/08/14	718	6.40	0.80	1.21	0.38	49	339
COLAIRIS	RE	08/26/14	506	2.80	0.81	0.60	0.23	26	192
COLAIRIS	RE	05/23/12	4	0.80	0.81	-	0.49	10	-
COLAIRIS	RE	07/25/13	52	3.50	0.82	0.35	0.04	12	61
COLAIRIS	RE	05/29/13	149	4.40	0.90	0.48	0.25	21	130
COLAIRIS	RE	05/14/11	105	1.80	0.91	0.23	0.06	11	73
COLAIRIS	RE	07/11/13	121	2.50	0.94	0.29	-	12	66
COLAIRIS	RE	07/15/13	73	1.60	0.96	-	-	6	36
COLAIRIS	RE	09/14/11	178	3.10	0.99	0.36	0.22	14	153
COLAIRIS	RE	08/21/14	356	3.60	1.02	0.58	0.11	28	247
COLAIRIS	RE	05/23/14	78	1.90	1.03	0.26	0.09	9	51
COLAIRIS	RE	05/12/14	39	2.50	1.08	0.31	0.24	11	45
COLAIRIS	RE	09/11/12	64	3.80	1.08	-	0.35	14	84
COLAIRIS	RE	07/27/11	170	2.90	1.12	0.27	0.03	15	99
COLAIRIS	RE	05/22/14	479	4.40	1.33	0.70	0.05	30	216

Site ID	Land Use	Date of Sample	TSS (mg/L)	TKN (mg/L)	NO_2+NO_3 (mg/L)	TP (mg/L)	DP (mg/L)	Tot. Cu (µg/L)	Tot. Zinc (µg/L)
COLAIRIS	RE	09/30/14	986	6.70	1.35	0.87	0.14	45	92
COLAIRIS	RE	07/01/13	902	8.80	1.51	1.76	-	54	468
COLAIRIS	RE	07/19/11	36	3.20	1.66	1.62	1.23	27	-
COLAIRIS	RE	09/25/12	11	2.00	1.80	-	0.23	6	-
COLAIRIS	RE	08/29/14	136	1.70	2.57	0.21	0.09	10	48
CODEGRHE	RE	07/14/09	38	1.32	0.24	0.37	0.17	-	-
CODEGRHE	RE	05/13/04	157	-	0.54	0.45	-	-	-
CODEGRHE	RE	04/07/06	29	2.60	0.63	0.23	0.22	-	-
CODEGRHE	RE	05/30/01	45	-	0.63	0.71	0.19	-	-
CODEGRHE	RE	06/04/08	454	4.20	0.65	0.73	-	-	-
CODEGRHE	RE	05/02/08	47	2.22	0.69	0.23	0.37	-	-
CODEGRHE	RE	05/10/06	8	2.50	0.78	0.19	0.26	-	-
CODEGRHE	RE	03/16/00	210	-	0.79	0.22	0.17	-	-
CODEGRHE	RE	06/06/07	194	-	0.80	0.65	0.22	-	-
CODEGRHE	RE	05/18/01	300	-	0.94	0.25	0.13	-	-
CODEGRHE	RE	06/10/04	680	-	1.01	1.03	0.22	-	-
CODEGRHE	RE	04/27/09	172	4.48	1.03	0.45	0.24	-	-
CODEGRHE	RE	08/27/04	56	-	1.04	0.24	0.12	-	-
CODEGRHE	RE	09/12/06	113	4.10	1.23	0.17	0.19	-	-
CODEGRHE	RE	06/16/02	83	-	1.26	0.72	0.71	-	-
CODEGRHE	RE	05/16/03	814	-	1.27	0.92	0.22	-	-
CODEGRHE	RE	06/04/02	308	-	1.36	0.71	0.29	-	-
CODEGRHE	RE	06/26/04	276	-	1.41	0.64	-	-	-
CODEGRHE	RE	08/06/07	200	2.90	1.47	0.67	0.37	-	-
CODEGRHE	RE	04/03/08	422	3.36	1.48	0.72	0.16	-	-
CODEGRHE	RE	04/17/07	96	5.80	1.91	0.40	0.24	-	-
CODEGRHE	RE	05/13/02	279	-	1.91	0.64	0.35	-	-
CODEGRHE	RE	05/02/07	233	7.50	2.08	0.72	0.31	-	-
CODEGRHE	RE	09/21/06	8	4.45	2.46	-	0.26	-	-
CODEGRHE	RE	09/19/01	128	-	3.46	0.53	0.35	-	-
CODEGRHE	RE	06/17/03	150	-	-	0.51	0.50	-	-
CODEGRHE	RE	08/20/00	78	-	-	-	-	-	-
CODEGRHE	RE	08/19/04	56	-	-	-	-	-	-
CODEGRHE	RE	09/15/05	239	-	-	-	-	-	-
CODEGRRE	RE	07/28/09	28	1.56	0.01	0.61	0.46	-	-
CODEGRRE	RE	07/14/09	14	1.19	0.04	0.35	0.26	-	-
CODEGRRE	RE	06/06/07	323	-	0.40	0.85	0.17	-	-
CODEGRRE	RE	06/16/02	602	-	0.47	1.51	1.33	-	-
CODEGRRE	RE	05/02/08	88	2.24	0.48	0.43	0.34	-	-
CODEGRRE	RE	06/04/08	662	2.96	0.53	0.45	-	-	-
CODEGRRE	RE	08/27/04	54	-	0.88	0.25	0.15	-	-
CODEGRRE	RE	06/26/04	65	-	0.88	0.40	0.17	-	-
CODEGRRE	RE	03/16/00	81	-	0.95	0.40	0.31	-	-
CODEGRRE	RE	05/13/04	288	-	0.95	0.83	-	-	-
CODEGRRE	RE	04/07/06	170	5.30	1.02	0.40	0.25	-	-
CODEGRRE	RE	05/23/09	254	2.31	1.04	0.50	0.10	-	-
CODEGRRE	RE	05/16/03	1210	-	1.15	1.71	0.31	-	-
CODEGRRE	RE	05/10/06	73	3.70	1.24	0.30	0.20	-	-
CODEGRRE	RE	05/18/01	530	-	1.40	0.11	0.14	-	-
CODEGRRE	RE	04/17/07	85	4.10	1.48	0.27	0.22	-	-
CODEGRRE	RE	08/06/07	258	0.45	1.49	0.64	0.37	-	-

Site ID	Land Use	Date of Sample	TSS (mg/L)	TKN (mg/L)	NO_2+NO_3 (mg/L)	TP (mg/L)	DP (mg/L)	Tot. Cu (µg/L)	Tot. Zinc (µg/L)
CODEGRRE	RE	04/27/09	257	5.90	1.55	0.86	0.46	-	-
CODEGRRE	RE	04/03/08	616	3.45	1.60	0.82	0.44	-	-
CODEGRRE	RE	05/02/07	525	7.20	1.76	1.30	0.24	-	-
CODEGRRE	RE	09/12/06	7	4.00	2.09	0.16	0.17	-	-
CODEGRRE	RE	09/21/06	34	-	-	0.43		-	-
CODEGRRE	RE	08/19/04	54	-	-	-	-	-	-
COAUSHCR	RE	07/19/94	999	7.30	1.45	1.16	-	68	420
COAUSHCR	RE	06/22/94	140	10.50	3.16	1.03	-	12	130
COAUSHCR	RE	04/13/91	-	-	-	0.12	-	20	130
COAUSHCR	RE	08/03/91	72	-	-	0.16	-	50	150
COAUSHCR	RE	06/01/91	14	-	-	0.25	-	5	30
COAUSHCR	RE	07/23/91	-	-	-	0.25	-	50	40
COAUSHCR	RE	07/29/90	18	1.80	-	0.32	-	10	50
COAUSHCR	RE	04/30/91	-	-	-	0.34	-	20	60
COAUSHCR	RE	07/21/91	30	-	-	0.34	-	100	30
COAUSHCR	RE	07/28/90	-	0.80	-	0.36	-	40	50
COAUSHCR	RE	07/05/90	164	2.30	-	0.37	-	50	110
COAUSHCR	RE	06/22/91	20	-	-	0.37	-	30	60
COAUSHCR	RE	05/29/90	72	1.50	-	0.39	-	10	50
COAUSHCR	RE	09/05/90	-	2.90	-	0.39	-	40	100
COAUSHCR	RE	09/19/90	-	1.80	-	0.44	-	30	-
COAUSHCR	RE	07/08/90	292	2.00	-	0.47	-	30	130
COAUSHCR	RE	07/10/91	-	-	-	0.47	-	60	50
COAUSHCR	RE	07/07/90	-	2.70	-	0.47	-	10	60
COAUSHCR	RE	05/23/91	-	-	-	0.54	-	30	140
COAUSHCR	RE	06/19/90	122	3.00	-	0.54	-	60	10
COAUSHCR	RE	08/04/90	-	2.70	-	0.71	-	40	190
COAUSHCR	RE	07/14/90	656	4.10	-	0.87	-	50	200
COAUSHCR	RE	08/17/90	352	2.50	-	1.08	-	30	60
COAUSHCR	RE	06/01/92	-	-	-	-	-	60	140
COAUSHCR	RE	06/05/92	-	-	-	-	-	-	-
COAUSHCR	RE	07/02/92	88	-	-	-	-	30	200
COAUSHCR	RE	07/12/92	44	-	-	-	-	10	190
COAUSHCR	RE	07/15/92	294	-	-	-	-	80	240
COAUSHCR	RE	07/17/92	-	-	-	-	-	30	80
COAUSHCR	RE	07/20/92	-	-	-	-	-	30	100
COAUSHCR	RE	07/22/92	-	-	-	-	-	10	80
COAUSHCR	RE	08/23/92	-	-	-	-	-	80	80
COAUSHCR	RE	06/22/96	221	12.20	0.08	0.61	-	20	220
COAUSHCR	RE	04/10/95	306	1.80	0.55	0.43	-	5	3
COAUSHCR	RE	09/20/95	40	1.10	0.76	0.22	-	9	40
COAUSHCR	RE	04/18/95	153	3.70	0.93	0.53	-	5	3
COAUSHCR	RE	09/11/96	521	1.40	1.04	0.11	-	16	170
COAUSHCR	RE	05/09/96	13	1.00	1.12	0.12	-	5	3
COAUSHCR	RE	06/15/96	501	5.70	1.32	0.71	-	5	160
COAUSHCR	RE	09/19/95	61	3.20	1.62	0.51	-	10	3
COAUSHCR	RE	04/23/95	9	0.50	1.63	0.08	-	5	3
COAUSHCR	RE	07/13/95	175	11.60	1.68	1.00	-	130	590
COAUSHCR	RE	04/23/95	389	4.60	1.68	1.83	-	5	3
COAUSHCR	RE	05/25/96	150	3.20	1.71	0.46	-	39	140
COAUSHCR	RE	10/22/95	124	10.10	1.77	1.04	-	20	130

Site ID	Land Use	Date of Sample	TSS (mg/L)	TKN (mg/L)	NO_2+NO_3 (mg/L)	TP (mg/L)	DP (mg/L)	Tot. Cu (µg/L)	Tot. Zinc (µg/L)
COAUSHCR	RE	10/04/95	59	1.60	1.78	0.25	-	5	80
COAUSHCR	RE	08/23/96	438	6.90	1.81	0.81	-	31	250
COAUSHCR	RE	06/13/95	324	6.20	1.82	0.75	-	26	200
COAUSHCR	RE	09/06/96	128	3.90	1.85	0.45	-	23	110
COAUSHCR	RE	09/29/95	203	3.00	2.16	0.10	-	23	150
COAUSHCR	RE	08/07/96	-	5.50	2.33	0.83	-	30	280
COAUSHCR	RE	08/18/95	182	4.40	2.85	0.58	-	17	110
COAUSHCR	RE	05/16/95	76	2.00	3.23	0.18	-	11	60
COAUSHCR	RE	07/09/96	4	2.00	6.00	0.19	-	4	40
COAUSHCR	RE	06/28/95	103	0.30	8.32	0.20	-	5	3
CODEORPO	RE	07/10/10	144	7.10	0.06	0.42	0.04	16	96
CODEORPO	RE	06/26/10	226	9.40	0.06	0.68	0.06	21	119
CODEORPO	RE	05/31/07	494	11.60	0.16	**	0.09	20	80
CODEORPO	RE	07/13/09	27	-	0.19	0.38	0.16	8	-
CODEORPO	RE	08/04/04	152	5.80	0.20	0.51	0.16	25	130
CODEORPO	RE	07/06/11	103	3.20	0.26	0.41	0.06	10	-
CODEORPO	RE	07/25/09	35	-	0.27	0.74	0.54	11	-
CODEORPO	RE	04/26/10	26	1.30	0.31	0.14	0.10	3	-
CODEORPO	RE	09/12/06	66	4.80	0.31	0.28	0.12	33	130
CODEORPO	RE	08/05/05	9	1.90	0.37	0.25	0.24	3	30
CODEORPO	RE	09/14/11	52	1.70	0.42	0.27	0.17	5	-
CODEORPO	RE	07/14/11	42	1.20	0.45	0.13	0.04	-	-
CODEORPO	RE	08/10/10	9	3.20	0.47	0.47	0.41	4	-
CODEORPO	RE	07/07/11	180	1.40	0.48	0.43	0.08	10	53
CODEORPO	RE	04/26/11	176	3.90	0.48	0.63	0.36	13	70
CODEORPO	RE	04/16/09	91	-	0.51	0.31	0.16	-	-
CODEORPO	RE	04/23/10	32	1.20	0.53	0.37	0.32	4	-
CODEORPO	RE	05/30/01	490	-	0.53	0.67	0.22	-	-
CODEORPO	RE	04/07/06	18	1.10	0.54	0.21	0.26	14	80
CODEORPO	RE	05/11/11	40	2.90	0.55	0.41	0.30	27	35
CODEORPO	RE	05/02/07	26	2.80	0.58	0.28	0.12	8	-
CODEORPO	RE	05/11/10	64	2.50	0.59	0.28	0.17	8	35
CODEORPO	RE	06/11/05	26	1.30	0.60	0.21	0.15	7	60
CODEORPO	RE	05/31/05	35	-	0.61	0.20	0.17	9	50
CODEORPO	RE	06/27/10	120	3.10	0.61	0.30	0.04	12	63
CODEORPO	RE	05/08/08	71	1.78	0.62	0.29	0.18	10	70
CODEORPO	RE	05/14/11	39	1.40	0.62	0.20	0.09	5	31
CODEORPO	RE	06/03/09	22	1.75	0.63	0.15	0.09	-	-
CODEORPO	RE	07/04/10	34	3.80	0.64	0.26	0.05	9	45
CODEORPO	RE	05/18/11	58	1.80	0.65	0.22	0.12	5	24
CODEORPO	RE	05/15/08	34	1.36	0.66	0.23	0.17	-	20
CODEORPO	RE	05/13/10	21	1.00	0.67	0.14	0.08	5	28
CODEORPO	RE	06/20/11	7	1.60	0.70	0.31	0.23	5	-
CODEORPO	RE	08/23/10	182	2.00	0.70	0.43	0.15	17	81
CODEORPO	RE	06/08/10	338	9.10	0.71	0.83	0.08	23	151
CODEORPO	RE	07/26/05	64	2.40	0.72	0.19	0.10	20	120
CODEORPO	RE	03/16/00	44	-	0.75	0.38	-	-	-
CODEORPO	RE	05/13/04	513	-	0.76	0.48	0.19	17	-
CODEORPO	RE	04/11/08	23	0.99	0.77	0.12	0.08	5	18
CODEORPO	RE	06/04/08	266	1.33	0.77	0.31	0.07	-	-
CODEORPO	RE	06/04/02	498	3.10	0.78	0.73	0.25	24	157

Site ID	Land Use	Date of Sample	TSS (mg/L)	TKN (mg/L)	NO_2+NO_3 (mg/L)	TP (mg/L)	DP (mg/L)	Tot. Cu (µg/L)	Tot. Zinc (µg/L)
CODEORPO	RE	06/25/09	49	-	0.79	0.44	0.30	16	-
CODEORPO	RE	07/27/09	35	-	0.79	0.37	0.16	6	-
CODEORPO	RE	06/06/07	112	-	0.80	0.62	0.11	-	-
CODEORPO	RE	06/07/07	103	7.30	0.80	-	0.15	31	80
CODEORPO	RE	07/10/06	12	3.60	0.81	0.51	0.47	-	-
CODEORPO	RE	05/05/06	-	3.60	0.81	-	0.16	-	-
CODEORPO	RE	06/26/06	12	3.60	0.81	-	0.47	8	40
CODEORPO	RE	07/22/10	140	2.50	0.81	0.30	0.06	12	81
CODEORPO	RE	07/11/01	398	2.80	0.85	0.58	0.61	25	126
CODEORPO	RE	05/14/10	342	3.00	0.85	0.35	0.08	12	79
CODEORPO	RE	08/27/04	320	-	0.88	0.52	0.18	17	-
CODEORPO	RE	08/23/07	648	3.10	0.88	-	0.16	13	57
CODEORPO	RE	06/17/03	216	2.80	0.91	0.40	0.39	7	128
CODEORPO	RE	04/22/10	38	1.60	0.94	0.20	0.12	7	111
CODEORPO	RE	08/08/10	33	4.00	0.95	0.75	0.64	9	26
CODEORPO	RE	08/09/10	33	4.00	0.95	0.75	0.63	10	37
CODEORPO	RE	09/13/02	209	2.40	0.99	0.30	0.14	16	59
CODEORPO	RE	09/15/04	107	2.40	0.99	0.32	0.48	16	70
CODEORPO	RE	06/23/09	5	-	0.99	1.73	1.62	9	-
CODEORPO	RE	06/28/04	337	-	1.02	0.53	0.19	7	-
CODEORPO	RE	07/20/09	17	-	1.02	0.97	0.86	8	-
CODEORPO	RE	07/12/11	18	1.10	1.03	0.19	0.12	-	-
CODEORPO	RE	06/12/10	19	1.50	1.05	0.21	0.14	5	-
CODEORPO	RE	04/17/07	28	3.00	1.05	0.38	0.17	6	-
CODEORPO	RE	07/03/02	85	2.10	1.06	0.45	0.32	5	38
CODEORPO	RE	06/01/09	208	-	1.07	0.95	0.37	31	170
CODEORPO	RE	06/12/10	25	1.50	1.07	0.24	0.18	6	-
CODEORPO	RE	06/11/10	94	1.50	1.07	0.32	0.18	6	30
CODEORPO	RE	05/23/09	146	2.72	1.08	0.52	0.11	-	-
CODEORPO	RE	06/26/08	124	2.09	1.10	0.48	0.09	26	150
CODEORPO	RE	04/03/08	24	1.71	1.11	0.24	0.16	-	-
CODEORPO	RE	05/09/09	75	-	1.12	0.76	0.41	17	62
CODEORPO	RE	04/24/11	52	2.70	1.14	0.37	0.26	-	-
CODEORPO	RE	04/28/11	52	2.70	1.14	0.37	0.26	9	40
CODEORPO	RE	04/17/08	9	2.01	1.14	0.21	0.17	74	12
CODEORPO	RE	05/10/06	128	3.60	1.15	0.36	0.22	-	-
CODEORPO	RE	06/10/04	1280	-	1.15	1.12	0.12	27	-
CODEORPO	RE	09/14/01	98	3.40	1.17	0.15	0.16	10	38
CODEORPO	RE	05/18/01	415	-	1.17	0.18	0.14	-	-
CODEORPO	RE	08/02/10	16	2.80	1.22	0.52	0.43	-	-
CODEORPO	RE	08/19/10	221	5.20	1.22	0.69	0.15	-	-
CODEORPO	RE	09/17/01	124	2.40	1.28	0.51	0.32	14	62
CODEORPO	RE	09/19/01	124	-	1.28	0.51	0.32	-	-
CODEORPO	RE	08/06/10	104	3.50	1.37	0.89	0.78	7	-
CODEORPO	RE	07/28/10	104	3.50	1.37	0.89	0.78	-	-
CODEORPO	RE	07/20/10	59	2.40	1.38	0.27	0.08	11	84
CODEORPO	RE	04/26/09	72	-	1.50	0.25	0.13	12	63
CODEORPO	RE	05/16/03	692	5.30	1.52	0.90	0.27	37	240
CODEORPO	RE	05/11/02	132	5.90	1.55	0.50	0.30	15	57
CODEORPO	RE	07/28/03	486	4.10	1.59	0.56	0.15	20	140
CODEORPO	RE	06/16/02	67	4.50	1.60	0.54	0.56	13	68

Site ID	Land Use	Date of Sample	TSS (mg/L)	TKN (mg/L)	NO_2+NO_3 (mg/L)	TP (mg/L)	DP (mg/L)	Tot. Cu (µg/L)	Tot. Zinc (µg/L)
CODEORPO	RE	06/26/04	252	-	1.76	0.94	0.23	15	-
CODEORPO	RE	07/27/10	88	3.50	1.94	0.32	0.05	10	68
CODEORPO	RE	08/06/07	10	4.60	2.12	0.36	0.32	15	-
CODEORPO	RE	09/21/06	7	2.70	2.13	0.47	0.53	-	-
CODEORPO	RE	09/21/06	8	2.70	2.13	**	0.53	6	30
CODEORPO	RE	04/21/10	94	13.10	2.13	0.82	0.55	34	-
CODEORPO	RE	08/15/07	138	7.12	2.21	-	0.89	30	113
CODEORPO	RE	04/28/06	13	1.60	2.65	0.37	0.31	-	-
CODEORPO	RE	05/02/08	512	2.80	2.70	1.25	0.39	-	70
CODEORPO	RE	06/25/09	49	-	-	0.44	0.30	-	-
CODEORPO	RE	05/31/09	208	-	-	0.95	0.37	-	-
CODEORPO	RE	05/25/07	-	-	-	**	**	-	-
COACWWL3	COM	04/10/08	302	-	-	0.50	0.02	-	-
COACWWL3	COM	04/17/08	6	-	-	0.07	0.01	-	-
COACWWL3	COM	05/02/08	154	-	-	0.23	0.08	-	-
COACWWL3	COM	05/13/08	16	-	-	0.07	0.03	-	-
COACWWL3	COM	05/15/08	33	-	-	0.08	0.04	-	-
COACWWL3	COM	05/27/08	33	-	-	0.12	0.04	-	-
COACWWL3	COM	06/04/08	123	-	-	0.12	0.01	-	-
COACWWL3	COM	07/09/08	82	-	-	0.14	0.04	-	-
COACWWL3	COM	08/12/08	137	-	-	0.35	0.08	-	-
COACWWL3	COM	08/15/08	77	-	-	0.24	0.12	-	-
COACWWL3	COM	09/12/08	44	-	-	0.14	0.07	-	-
COACWWL3	COM	10/06/08	141	-	-	0.54	0.12	-	-
COACWWL3	COM	10/13/08	11	-	-	0.16	0.03	-	-
COACWWL3	COM	04/17/09	9	-	-	0.01	-	-	-
COACWWL3	COM	04/26/09	37	-	-	0.09	0.04	-	-
COACWWL3	COM	05/08/09	13	-	-	0.04	-	-	-
COACWWL3	COM	05/20/09	4	-	-	0.01	0.01	-	-
COACWWL3	COM	05/25/09	90	-	-	0.11	0.01	-	-
COACWWL3	COM	06/01/09	95	-	-	0.17	0.03	-	-
COACW6W7	COM	04/10/08	19	-	-	0.30	0.21	-	-
COACW6W7	COM	04/17/08	14	-	-	0.20	0.16	-	-
COACW6W7	COM	05/02/08	186	-	-	0.32	0.19	-	-
COACW6W7	COM	05/13/08	13	-	-	0.15	0.13	-	-
COACW6W7	COM	05/15/08	8	-	-	0.12	0.10	-	-
COACW6W7	COM	06/20/08	69	-	-	0.40	0.21	-	-
COACW6W7	COM	07/09/08	57	-	-	0.19	0.14	-	-
COACW6W7	COM	08/07/08	14	-	-	0.35	0.24	-	-
COACW6W7	COM	08/12/08	51	-	-	0.32	0.23	-	-
COACW6W7	COM	08/15/08	21	-	-	0.36	0.30	-	-
COACW6W7	COM	09/12/08	16	-	-	0.32	0.29	-	-
COACW6W7	COM	04/17/09	58	-	-	0.39	-	-	-
COACW6W7	COM	04/26/09	40	-	-	0.17	0.10	-	-
COACW6W7	COM	05/08/09	35	-	-	0.32	-	-	-
COACW6W7	COM	05/25/09	21	-	-	0.17	0.12	-	-
COACW6W7	COM	05/31/09	22	-	-	0.18	0.13	-	-
COACW6W7	COM	06/01/09	21	-	-	0.11	0.08	-	-
CODEWAWA	COM	08/08/08	1360	4.50	0.19	0.90	0.04	32	188
CODEWAWA	COM	08/15/08	131	1.20	-	0.13	0.04	-	-
CODEWAWA	COM	09/11/08	6	4.20	-	0.46	0.02	-	-

Site ID	Land Use	Date of Sample	TSS (mg/L)	TKN (mg/L)	NO_2+NO_3 (mg/L)	TP (mg/L)	DP (mg/L)	Tot. Cu (µg/L)	Tot. Zinc (µg/L)
CODEWAWA	COM	04/16/09	129	3.00	0.96	0.32	0.12	-	-
CODEWAWA	COM	05/22/09	5	4.70	0.72	0.48	0.14	-	-
CODEWAWA	COM	05/24/09	436	2.50	0.19	0.38	0.05	-	-
CODEWAWA	COM	06/02/09	116	1.80	0.73	0.15	0.03	-	-
CODEWAWA	COM	06/13/09	104	2.20	0.64	0.14	0.03	-	-
CODEWAWA	COM	06/25/09	235	-	-	-	-	-	-
CODEWAWA	COM	06/26/09	230	4.10	0.05	0.36	0.09	-	-
CODEWAWA	COM	07/01/09	91	1.80	0.48	0.19	0.10	-	-
CODEWAWA	COM	07/03/09	55	2.00	0.34	0.14	0.06	-	-
CODEWAWA	COM	07/20/09	481	4.20	0.15	0.65	0.18	-	-
CODEWAWA	COM	07/25/09	427	1.80	0.18	0.26	0.03	-	-
CODEWAWA	COM	07/26/09	80	2.30	0.22	0.32	0.15	-	-
CODEWAWA	COM	07/29/09	78	1.80	0.61	0.10	0.04	-	-
CODEWAWA	COM	08/09/09	141	2.40	0.43	0.19	0.07	-	-
CODEWAWA	COM	04/22/10	886	3.70	0.29	0.72	0.09	-	-
CODEWAWA	COM	04/23/10	301	1.40	0.05	0.28	0.03	-	-
CODEWAWA	COM	05/11/10	91	2.10	0.33	0.34	0.20	-	-
CODEWAWA	COM	05/14/10	202	1.60	0.55	0.17	0.01	-	-
CODEWAWA	COM	06/12/10	147	2.00	0.11	0.28	0.06	-	-
CODEWAWA	COM	07/04/10	427	2.80	0.09	0.48	0.04	-	-
CODEWAWA	COM	07/07/10	384	2.80	0.31	0.49	0.17	-	-
CODEWAWA	COM	07/20/10	414	6.70	0.08	1.32	0.47	-	-
CODEWAWA	COM	08/24/10	102	3.10	0.81	0.37	0.18	-	-
CODEWAWA	COM	05/11/11	327	6.50	0.99	0.78	0.43	26	158
CODEWAWA	COM	05/14/11	64	2.00	0.85	0.17	0.03	13	52
CODEWAWA	COM	05/18/11	546	4.10	0.83	0.74	0.03	54	326
CODEWAWA	COM	05/19/11	323	2.20	0.41	0.35	0.04	25	165
CODEWAWA	COM	05/24/11	1310	3.90	0.62	0.73	0.05	62	402
CODEWAWA	COM	06/13/11	1730	12.40	0.86	2.31	0.08	165	896
CODEWAWA	COM	06/20/11	571	4.40	0.56	0.49	0.05	48	276
CODEWAWA	COM	07/07/11	-	4.80	0.07	1.65	0.03	83	495
CODEWAWA	COM	07/10/11	221	2.30	0.95	0.36	0.05	21	72
CODEWAWA	COM	07/11/11	74	1.40	0.55	0.22	0.07	14	52
CODEWAWA	COM	07/12/11	585	2.80	0.93	0.70	0.04	34	185
CODEWAWA	COM	07/13/11	1540	3.20	0.13	1.13	0.03	63	371
CODEWAWA	COM	07/14/11	190	1.10	0.62	0.12	0.03	14	-
CODEWAWA	COM	07/19/11	238	3.40	1.16	0.44	0.06	27	135
CODEWAWA	COM	07/26/11	180	3.00	0.84	0.36	0.06	25	111
CODEWAWA	COM	08/01/11	355	4.10	0.59	0.64	0.07	38	215
CODEWAWA	COM	09/06/11	69	11.30	3.44	0.77	0.56	75	155
CODEWAWA	COM	09/07/11	59	3.00	0.90	0.24	0.14	26	55
CODEWAWA	COM	09/14/11	72	2.20	0.61	0.21	0.12	17	61
CODEWAWA	COM	07/07/13	877	8.50	0.41	1.48	-	93	529
CODEWAWA	COM	07/11/13	77	2.60	0.35	0.51	-	18	65
CODEWAWA	COM	07/12/13	160	4.50	0.35	0.44	-	34	92
CODEWAWA	COM	07/27/13	280	4.00	-	0.40	0.13	31	125
CODEWAWA	COM	08/12/13	25	3.50	-	0.38	0.25	24	35
CODEWAWA	COM	09/11/13	11	1.10	-	0.12	0.05	8	-
CODEWAWA	COM	09/22/13	68	2.70	-	0.26	0.18	17	50
CODEWAWA	COM	05/08/14	2260	23.90	3.61	4.44	1.59	224	1440
CODEWAWA	COM	05/09/14	160	2.90	0.58	0.51	0.18	34	171

Site ID	Land Use	Date of Sample	TSS (mg/L)	TKN (mg/L)	NO_2+NO_3 (mg/L)	TP (mg/L)	DP (mg/L)	Tot. Cu (µg/L)	Tot. Zinc (µg/L)
CODEWAWA	COM	05/12/14	87	2.30	2.14	0.30	0.08	18	88
CODEWAWA	COM	05/22/14	1240	6.80	0.35	1.24	0.06	101	649
CODEWAWA	COM	05/23/14	1590	5.60	0.34	1.52	0.03	104	624
CODEWAWA	COM	06/19/14	649	3.00	0.42	0.49	0.09	34	196
CODEWAWA	COM	07/08/14	696	5.40	0.68	0.84	0.07	62	375
CODEWAWA	COM	07/14/14	226	3.30	0.08	0.41	0.07	33	180
CODEWAWA	COM	07/17/14	41	2.20	0.51	0.14	0.03	14	42
CODEWAWA	COM	07/24/14	345	3.10	0.87	0.45	0.06	34	170
CODEWAWA	COM	07/30/14	437	2.50	1.10	0.39	0.03	34	189
CODEWAWA	COM	08/27/14	358	2.10	0.47	0.45	0.04	35	234
CODEWAWA	COM	08/28/14	171	1.40	0.55	0.23	0.05	21	111
CODEWAWA	COM	09/05/14	15	1.60	0.63	0.16	0.08	21	76
CODEWAWA	COM	09/10/14	39	1.50	0.53	0.19	0.14	53	74
COLASHOP	COM	06/04/05	-	1.00	0.43	0.17	-	-	-
COLASHOP	COM	06/09/05	-	1.00	0.43	0.17	-	-	-
COLASHOP	COM	06/12/05	13	0.30	1.72	0.02	-	-	-
COLASHOP	COM	06/20/05	85	1.50	0.40	0.06	-	-	-
COLASHOP	COM	06/21/05	29	4.00	2.98	0.12	-	-	-
COLASHOP	COM	08/03/05	16	1.70	0.85	0.09	-	-	-
COLASHOP	COM	08/04/05	17	1.80	0.46	0.08	-	-	-
COLASHOP	COM	08/09/05	16	4.50	1.76	0.10	-	-	-
COLASHOP	COM	08/10/05	5	1.40	1.26	0.08	-	-	-
COLASHOP	COM	08/20/05	63	3.70	1.61	0.21	-	-	-
COLASHOP	COM	04/30/06	-	1.10	0.24	0.10	-	-	-
COLASHOP	COM	05/03/06	-	2.40	0.80	0.30	-	-	-
COLASHOP	COM	05/09/06	-	3.20	0.99	0.22	-	-	-
COLASHOP	COM	08/03/06	-	2.40	0.79	0.14	-	-	-
COLASHOP	COM	09/21/06	-	2.60	0.77	0.13	-	-	-
COLASHOP	COM	04/10/07	40	-	-	0.17	-	-	-
COLASHOP	COM	04/16/07	115	-	-	0.20	-	-	-
COLASHOP	COM	04/23/07	60	-	-	0.07	-	-	-
COLASHOP	COM	05/01/07	455	-	-	0.44	-	-	-
COLASHOP	COM	05/07/07	46	-	-	0.12	-	-	-
COLASHOP	COM	05/14/07	303	-	-	0.24	-	-	-
COLASHOP	COM	05/29/07	165	-	-	0.18	-	-	-
COLASHOP	COM	06/12/07	37	-	-	0.15	-	-	-
COLASHOP	COM	07/08/07	297	-	-	0.42	-	-	-
COLASHOP	COM	07/27/07	35	-	-	0.20	-	-	-
COLASHOP	COM	08/05/07	130	-	-	0.07	-	-	-
COLASHOP	COM	08/10/07	518	-	-	0.37	-	-	-
COLASHOP	COM	05/02/08	27	1.30	-	0.93	-	-	-
COLASHOP	COM	05/14/08	26	0.80	-	0.05	-	-	-
COLASHOP	COM	05/27/08	156	3.00	-	0.30	-	-	-
COLASHOP	COM	06/06/08	19	1.70	-	0.08	-	-	-
COLASHOP	COM	08/18/08	16	0.80	-	0.06	-	-	-
COLASHOP	COM	09/15/08	21	1.00	-	0.07	-	-	-
COLASHOP	COM	04/16/09	65	1.40	0.28	0.16	0.05	-	-
COLASHOP	COM	04/17/09	53	1.20	0.13	0.15	0.04	-	-
COLASHOP	COM	04/26/09	47	1.40	0.31	0.12	0.04	-	-
COLASHOP	COM	05/23/09	1020	4.80	0.73	0.06	0.97	-	-
COLASHOP	COM	05/25/09	29	0.60	0.12	0.05	0.02	-	-

Site ID	Land Use	Date of Sample	TSS (mg/L)	TKN (mg/L)	NO_2+NO_3 (mg/L)	TP (mg/L)	DP (mg/L)	Tot. Cu (µg/L)	Tot. Zinc (µg/L)
COLASHOP	COM	06/01/09	137	1.80	0.48	0.15	0.03	-	-
COLASHOP	COM	06/07/09	38	2.50	1.15	0.12	0.03	-	-
COLASHOP	COM	06/11/09	41	1.40	0.82	0.09	0.02	-	-
COLASHOP	COM	06/13/09	1180	1.70	0.33	0.34	0.02	-	-
COLASHOP	COM	06/14/09	539	1.20	0.56	0.22	0.03	-	-
COLASHOP	COM	06/23/09	39	2.60	1.14	0.15	0.05	-	-
COLASHOP	COM	06/26/09	-	1.30	0.73	0.14	0.02	-	-
COLASHOP	COM	06/26/09	190	-	-	-	-	-	-
COLASHOP	COM	07/01/09	156	1.40	0.38	0.14	0.04	-	-
COLASHOP	COM	07/03/09	347	2.10	0.69	0.26	0.02	-	-
COLASHOP	COM	07/04/09	50	1.40	0.32	0.05	0.02	-	-
COLASHOP	COM	07/10/09	59	2.30	1.31	0.12	0.02	-	-
COLASHOP	COM	07/20/09	465	2.80	0.30	0.58	0.14	-	-
COLASHOP	COM	07/25/09	7	0.70	0.03	0.16	0.08	-	-
COLASHOP	COM	07/27/09	293	1.50	0.45	0.24	0.05	-	-
COLASHOP	COM	07/29/09	20	1.40	0.34	0.12	0.05	-	-
COLASHOP	COM	08/06/09	94	2.40	1.27	0.14	-	-	-
COLASHOP	COM	08/09/09	326	1.20	0.42	0.19	0.07	-	-
COLASHOP	COM	08/17/09	164	2.40	0.64	0.16	0.08	-	-
COLASHOP	COM	08/18/09	31	1.60	0.69	-	-	-	-
COLASHOP	COM	09/12/09	32	4.40	1.64	0.31	0.19	-	-
COLASHOP	COM	09/23/09	15	1.60	0.78	0.23	0.18	-	-
COLASHOP	COM	04/21/10	620	3.10	0.45	0.49	-	-	-
COLASHOP	COM	04/22/10	135	0.70	0.15	0.14	0.04	-	-
COLASHOP	COM	04/23/10	163	0.50	0.04	0.11	0.01	-	-
COLASHOP	COM	05/11/10	264	2.40	0.48	0.36	0.08	-	-
COLASHOP	COM	05/12/10	80	1.70	0.46	0.16	0.05	-	-
COLASHOP	COM	05/13/10	39	0.80	0.49	0.12	0.06	-	-
COLASHOP	COM	05/13/10	1940	3.70	1.45	0.41	0.04	-	-
COLASHOP	COM	06/11/10	1510	4.10	0.62	0.65	0.03	-	-
COLASHOP	COM	06/13/10	52	0.50	0.14	0.07	0.02	-	-
COLASHOP	COM	06/26/10	149	8.90	1.92	0.33	0.04	-	-
COLASHOP	COM	07/04/10	571	5.20	0.68	0.50	0.03	-	-
COLASHOP	COM	07/06/10	303	2.20	0.36	0.26	0.03	-	-
COLASHOP	COM	07/19/10	203	5.70	0.90	0.40	0.04	-	-
COLASHOP	COM	07/20/10	61	2.30	1.27	0.14	0.02	-	-
COLASHOP	COM	07/22/10	127	1.80	0.94	0.17	0.03	-	-
COLASHOP	COM	08/09/10	134	2.50	0.88	0.23	0.07	-	-
COLASHOP	COM	04/18/11	125	2.40	0.50	0.26	0.05	12	81
COLASHOP	COM	05/10/11	525	3.30	0.22	0.41	0.04	18	137
COLASHOP	COM	05/10/11	30	0.70	0.17	0.06	0.05	7	21
COLASHOP	COM	05/18/11	757	2.40	0.41	0.80	0.00	35	215
COLASHOP	COM	05/19/11	42	0.40	0.18	0.05	0.00	40	23
COLASHOP	COM	06/19/11	234	1.80	0.21	0.26	0.04	12	93
COLASHOP	COM	06/19/11	50	1.20	0.30	0.07	0.00	10	46
COLASHOP	COM	07/07/11	763	2.10	0.53	0.36	0.03	20	117
COLASHOP	COM	07/12/11	219	0.90	0.20	0.66	0.04	12	53
COLASHOP	COM	07/13/11	243	1.30	0.22	0.15	0.00	8	0
COLASHOP	COM	07/19/11	184	1.20	0.72	0.23	0.00	9	56
COLASHOP	COM	07/26/11	55	1.20	0.53	0.10	0.06	5	0
COLASHOP	COM	07/27/11	96	2.40	0.80	0.11	0.03	4	0

Site ID	Land Use	Date of Sample	TSS (mg/L)	TKN (mg/L)	NO_2+NO_3 (mg/L)	TP (mg/L)	DP (mg/L)	Tot. Cu (µg/L)	Tot. Zinc (µg/L)
COLASHOP	COM	09/11/12	7	0.60	0.18	0.06	-	4	0
COLASHOP	COM	09/25/12	8	1.40	0.33	0.13	-	4	0
COLASHOP	COM	04/13/13	156	1.80	0.37	0.21	0.07	-	-
COLASHOP	COM	05/07/13	129	3.40	0.83	0.21	0.08	11	64
COLASHOP	COM	05/08/13	58	1.30	0.34	0.17	0.08	7	34
COLASHOP	COM	05/20/13	35	1.80	0.72	0.13	0.05	9	26
COLASHOP	COM	05/29/13	38	1.30	0.32	0.11	0.06	-	-
COLASHOP	COM	07/01/13	22	3.20	0.97	0.17	-	10	45
COLASHOP	COM	07/06/13	134	2.30	0.53	0.15	-	12	73
COLASHOP	COM	07/11/13	308	2.50	0.63	0.31	-	16	107
COLASHOP	COM	07/13/13	226	1.00	0.31	0.16	-	5	37
COLASHOP	COM	09/09/13	87	2.00	0.76	0.15	0.06	15	75
COLASHOP	COM	09/10/13	7	0.80	1.17	0.02	0.05	3	0
COLASHOP	COM	09/22/13	16	0.50	0.18	0.05	0.04	2	0
COLASHOP	COM	05/12/14	56	0.90	0.30	0.12	0.00	5	32
COLASHOP	COM	05/23/14	193	1.90	0.81	0.24	0.02	16	103
COLASHOP	COM	05/31/14	672	2.80	0.49	0.45	0.03	26	191
COLASHOP	COM	06/09/14	192	1.50	0.32	0.26	-	11	64
COLASHOP	COM	06/19/14	64	2.70	0.96	0.24	0.05	9	31
COLASHOP	COM	07/04/14	18	3.70	0.08	0.14	0.03	11	39
COLASHOP	COM	07/17/14	23	1.30	0.41	0.08	0.03	4	0
COLASHOP	COM	07/30/14	10	0.00	0.31	0.05	0.11	3	0
COLASHOP	COM	07/30/14	9	0.40	0.17	0.02	0.02	2	0
COLASHOP	COM	08/08/14	9	1.50	0.65	0.06	0.01	7	0
COLASHOP	COM	08/21/14	13	2.30	1.10	0.15	0.10	8	21
COLASHOP	COM	08/27/14	82	0.90	0.51	0.06	0.01	6	14
COLASHOP	COM	08/28/14	12	0.60	0.33	0.04	0.03	4	10
COLASHOP	COM	09/05/14	8	0.90	0.54	0.07	0.05	8	19
COLASHOP	COM	09/22/14	16	1.00	0.23	0.07	0.06	4	26
COLASHOP	COM	09/29/14	66	2.00	0.50	0.11	0.02	8	44
COLASHOP	COM	09/30/14	106	1.30	0.45	0.11	0.02	10	51
COLASHOP	COM	10/10/14	47	1.30	0.38	0.07	0.06	6	49
COLAMOP4	COM	07/15/04	4	1.10	1.82	0.08	-	-	-
COLAMOP4	COM	07/16/04	17	1.10	1.01	0.10	-	-	-
COLAMOP4	COM	07/22/04	4	0.70	2.22	0.05	-	-	-
COLAMOP4	COM	08/18/04	46	1.10	0.59	0.08	-	-	-
COLAMOP4	COM	08/27/04	4	0.70	1.29	0.07	-	-	-
COLAMOP4	COM	09/27/04	12	0.70	1.07	0.06	-	-	-
COLAMOP4	COM	10/05/04	5	1.00	0.49	0.05	-	-	-
COLAMOP6	COM	08/23/05	5	1.40	1.26	0.08	-	-	-
COLAMOP6	COM	04/28/06	16	2.60	0.77	0.13	-	-	-
COLAMOP6	COM	06/24/06	111	2.40	0.80	0.30	-	-	-
COLAMOP6	COM	08/03/06	17	2.40	0.79	0.14	-	-	-
COLAMOP6	COM	09/22/06	16	2.60	0.77	0.13	-	-	-
COLAMOP4	COM	04/16/95	32	3.10	1.29	0.11	-	-	-
COLAMOP4	COM	04/17/95	36	0.70	0.91	0.09	-	-	-
COLAMOP4	COM	04/29/95	27	1.50	0.67	0.13	-	-	-
COLAMOP4	COM	05/16/95	11	0.60	0.15	0.01	-	-	-
COLAMOP4	COM	06/17/95	22	1.10	0.17	0.04	-	-	-
COLAMOP4	COM	07/13/95	88	2.80	1.04	0.18	-	-	-
COLAMOP4	COM	08/18/95	175	2.50	1.14	0.12	-	-	-

Site ID	Land Use	Date of Sample	TSS (mg/L)	TKN (mg/L)	NO_2+NO_3 (mg/L)	TP (mg/L)	DP (mg/L)	Tot. Cu (µg/L)	Tot. Zinc (µg/L)
COLAMOP4	COM	04/27/96	10	1.10	0.70	0.01	-	-	-
COLAMOP4	COM	05/29/96	41	13.00	0.49	0.11	-	-	-
COLAMOP4	COM	08/13/96	450	9.90	2.88	0.49	-	-	-
COLAMOP4	COM	08/16/96	126	9.90	1.13	0.17	-	-	-
COLAMOP4	COM	07/27/97	182	4.30	2.03	0.12	-	-	-
COLAMOP4	COM	07/28/97	57	1.40	0.36	0.04	-	-	-
COLAMOP4	COM	08/01/97	18	1.60	0.98	0.08	-	-	-
COLAMOP4	COM	08/04/97	18	0.90	0.40	0.03	-	-	-
COLAMOP4	COM	08/17/97	25	1.00	1.50	0.11	-	-	-
COLAMOP4	COM	09/22/97	14	0.80	0.95	0.03	-	-	-
COLAMOP4	COM	05/04/98	199	2.30	0.95	0.50	-	-	-
COLAMOP4	COM	05/08/98	218	1.30	0.63	0.38	-	-	-
COLAMOP4	COM	05/22/98	52	2.20	0.66	0.14	-	-	-
COLAMOP4	COM	06/04/98	11	2.10	0.63	0.09	-	-	-
COLAMOP4	COM	06/14/98	37	1.80	0.33	0.18	-	-	-
COLAMOP4	COM	07/08/98	20	2.30	1.05	0.26	-	-	-
COLAMOP4	COM	07/22/98	22	2.20	0.85	0.17	-	-	-
COLAMOP4	COM	07/23/98	21	1.20	0.43	0.14	-	-	-
COLAMOP4	COM	07/30/98	19	1.20	0.63	0.07	-	-	-
COLAMOP4	COM	08/10/98	26	0.90	1.20	0.07	-	-	-
COLAMOP4	COM	09/01/98	25	1.00	0.52	0.20	-	-	-
COFEMORR	OP	04/23/80	464	5.57	0.56	0.53	0.17	20	120
COFEMORR	OP	04/30/80	553	3.10	0.66	0.56	0.15	20	90
COFEMORR	OP	05/08/80	194	1.72	0.38	0.25	0.08	10	90
COFEMORR	OP	05/15/80	257	1.96	0.09	0.27	0.07	120	50
COFEMORR	OP	05/16/80	204	1.30	0.19	0.21	0.09	10	40
COFEMORR	OP	05/17/81	239	3.28	0.88	0.41	0.17	20	140
COFEMORR	OP	06/03/81	866	3.26	0.85	0.66	0.21	60	180

2
