# Supplementary material for: Predicting nonpoint stormwater runoff quality from land use
Source: PLoS One. 2018 May 9;13(5):e0196782. doi: 10.1371/journal.pone.0196782 (PMC5942771; doi:10.1371/journal.pone.0196782)
Supplement: S3 Table — (PDF) [file pone.0196782.s003.pdf]

S3 Table. Raw Sampling Data from NSQD for Validation using Mixed Land Use Sites

Site ID	Date	TSS (mg/L)	TKN (mg/L)	NO2+NO3 (mg/L as N)	TP (mg/L)	DP (mg/L)	Tot. Cu (ug/L)	Tot. Zinc (ug/L)
**CODEA001**	06/05/92	267	5.50	2.90	1.00	0.32	170	560
**CODEA001**	06/19/92	291	4.70	2.70	0.55	0.36	340	730
**CODEA001**	07/12/92	83	1.80	2.10	0.32	0.14	45	220
**CODEA004**	05/21/92	245	6.80	1.90	1.30	0.85	60	740
**CODEA004**	06/08/92	370	1.00	0.70	0.14	0.10	86	620
**CODEA004**	07/10/92	242	4.60	1.60	0.91	0.52	41	490
**CODEA006**	06/08/92	540	2.10	1.50	0.36	0.18	44	620
**CODEA006**	06/25/92	944	2.50	1.10	0.85	0.25	66	590
**CODEA006**	07/10/92	464	3.60	2.10	0.71	0.27	39	520
**COJENOAV**	05/08/80	656	2.28	1.59	0.69	0.12	50	270
**COJENOAV**	05/11/80	558	3.12	3.02	0.54	0.07	50	540
**COJENOAV**	05/12/80	546	2.20	2.15	0.49	0.04	40	420
**COJENOAV**	05/15/80	339	1.69	0.87	0.34	0.05	30	190
**COJENOAV**	05/17/80	432	1.70	1.30	0.43	0.06	30	280
**COJENOAV**	07/24/80	557	7.27	2.70	1.08	0.34	60	600
**COJENOAV**	08/10/80	564	10.99	3.80	0.80	0.35	60	640
**COJENOAV**	09/08/80	148	2.03	0.76	0.27	0.13	20	170
**COJENOAV**	09/10/80	128	-	1.00	0.24	0.09	20	150
**COJENOAV**	09/10/80	796	2.92	0.93	0.85	0.07	50	360
**COJENOAV**	03/03/81	380	-	1.40	0.57	0.27	-	720
**COJENOAV**	05/03/81	342	6.83	2.22	1.21	0.42	140	890
**COJENOAV**	05/05/81	568	4.90	2.00	1.80	0.46	90	580
**COJENOAV**	05/09/81	215	3.00	0.76	0.43	0.15	30	210
**COJENOAV**	05/16/81	354	4.30	1.10	0.69	0.21	60	340
**COJENOAV**	05/28/81	548	3.80	0.94	0.66	0.09	80	400
**COJENOAV**	05/28/81	392	2.50	0.78	0.53	0.09	50	260
**COJENOAV**	07/26/81	747	2.50	1.00	0.74	0.13	100	430
**COJENOAV**	08/03/81	-	16.56	6.02	3.06	1.30	750	3610
**COJENOAV**	08/12/81	532	2.90	0.09	0.72	-	60	330
