# Supplementary material for: Predicting nonpoint stormwater runoff quality from land use
Source: PLoS One. 2018 May 9;13(5):e0196782. doi: 10.1371/journal.pone.0196782 (PMC5942771; doi:10.1371/journal.pone.0196782)
Supplement: S4 Table — (PDF) [file pone.0196782.s004.pdf]

S4 Table. Statistical Analysis for All Constituents

Summary of Non-Point Runoff Data	TSS [mg/L]	TKN [mg/L]	NO_2+NO_3 [mg/L as N]	TP [mg/L]	DP [mg/L]	Cu [μg/L]	Zn [μg/L]
Residential							
**No. of Samples**	246	196	226	235	192	186	155
**Mean Conc.**	204 ± 18.5	3.41 ± 0.17	1.07 ± 0.05	0.51 ± 0.02	0.25 ± 0.01	20.1 ± 1.84	104.0 ± 11.6
**St. Deviation**	239	2.37	0.85	0.33	0.22	18.2	87.2
**Median Conc.**	120.5	2.85	0.91	0.43	0.18	14.3	80
**Max[1]**	1310	13.4	8.32	1.91	1.62	130	590
Commercial							
**No. of Samples**	261	220	209	267	172	85	83
**Mean Conc.**	193 ± 18.0	2.53 ± 0.16	0.70 ± 0.05	0.28 ± 0.02	0.09 ± 0.01	27.6 ± 2.7	143.1 ± 15.8
**St. Deviation**	333	2.44	0.56	0.38	0.16	34.8	216.9
**Median Conc.**	66	2.00	0.58	0.17	0.05	15.95	72.1
**Max**	2260	23.9	3.61	4.44	1.59	224	1440
Open Space							
**No. of Samples**	7	7	7	7	7	7	7
**Mean Conc.**	397 ± 109.9	2.88 ± 0.91	0.52 ± 0.27	0.41 ± 0.13	0.13 ± 0.07	37.1 ± 9.5	101.4 ± 54.6
**St. Deviation**	249	1.43	0.31	0.18	0.05	40.3	49.5
**Median Conc.**	257	3.10	0.56	0.41	0.15	20.0	90.0
**Max**	866	5.57	0.88	0.66	0.21	120.0	180.0
Summary of One-Tailed T-Test for Residential and Commercial Land Uses							
**P-value** $H_o: \mu_{res} = \mu_{com}$	0.33	1×10^{-4}	1×10^{-7}	1×10^{-13}	1×10^{-14}	0.03	0.06
**Null Hypothesis** $\alpha = 0.05$	Accept	Reject	Reject	Reject	Reject	Reject	Accept

[1] Maximum values are provided to show uncertainty with respect to the mean and median values as maxima have large influence on the results. Minimum values are not reported as data is either reported missing or a value of zero is reported. The value of zero signifies a value that was less than the Minimum Detect Level values that were not reported. Standard errors on the means were calculated based on the MATLAB script provided in the additional supplemental information using the FITLM function for each constituent in a multi-linear regression analysis. Additional information on regression, correlation, and other statistical analyses of the residential and commercial datasets can be found in Zivkovich (2015).
