# Supplementary material for: Predicting nonpoint stormwater runoff quality from land use
Source: PLoS One. 2018 May 9;13(5):e0196782. doi: 10.1371/journal.pone.0196782 (PMC5942771; doi:10.1371/journal.pone.0196782)
Supplement: S5 Table — (PDF) [file pone.0196782.s005.pdf]

S5 Table. Summary of Linear Correlations and Relationships between Similar Constituents in Developed Land Use Classes

LINEST Statistics	Variable	TKN vs. NO2+NO3		TP vs. DP		Zn vs. Cu	
		Res.	Com.	Res.	Com.	Res.	Com.
**Slope**	m	0.17	2.11	0.81	1.70	2.61	5.99
**Standard Error (Slope)**	se_{slope}	0.19	0.27	0.09	0.17	0.30	0.19
**Coefficient of Determination**	R^2	0.004	0.223	0.303	0.364	0.329	0.934
