# Supplementary material for: Predicting nonpoint stormwater runoff quality from land use
Source: PLoS One. 2018 May 9;13(5):e0196782. doi: 10.1371/journal.pone.0196782 (PMC5942771; doi:10.1371/journal.pone.0196782)
Supplement: S6 Table — (PDF) [file pone.0196782.s006.pdf]

S6 Table. Overview of Geospatial Analysis for Application to Lakewood Gulch Watershed

**Sub-Basins**	**Sub-Basin Area (km^2)**	**% of Total Area**	**Land Use 1 - Residential A**		**Land Use 2 - Residential B**		**Land Use 3 - Commercial**		**Developed Area Percentages**
			Area (km^2)	% of Sub-Basin	Area (km^2)	% of Sub-Basin	Area (km^2)	% of Sub-Basin	
**1**	3.5	6.8%	1.7	48.6%	0.8	22.5%	0.2	6.1%	77.2%
**2**	5.1	9.9%	2.7	52.5%	0.8	14.9%	0.2	4.2%	71.5%
**3**	7.4	14.5%	3.2	43.0%	1.6	22.1%	0.7	9.3%	74.4%
**4**	4.2	8.3%	1.8	42.3%	0.9	22.0%	0.5	12.3%	76.6%
**5**	9.7	19.0%	3.9	39.9%	2.3	23.3%	1.5	15.6%	78.8%
**6**	5.6	10.9%	2.7	48.6%	0.8	15.0%	0.1	2.5%	66.2%
**7**	9.4	18.5%	2.7	28.7%	1.6	17.4%	0.9	9.1%	55.2%
**8**	6.3	12.3%	2.0	31.4%	1.5	23.3%	0.5	8.5%	63.2%
**Mean**	6.4	13%	2.6	41.9%	1.3	20.1%	0.6	8.4%	70.4%
**Entire Watershed**	**51.1**	**100%**	**20.5**	**40.2%**	**10.3**	**20.1%**	**4.7**	**9.1%**	**69.6%**
