# Supplementary material for: Predicting nonpoint stormwater runoff quality from land use
Source: PLoS One. 2018 May 9;13(5):e0196782. doi: 10.1371/journal.pone.0196782 (PMC5942771; doi:10.1371/journal.pone.0196782)
Supplement: S1 File — This file includes a copyright permission letter from Environmental Systems Research Institute (ESRI) allowing permission to publish the basemap used with Figs 3 and 4. (PDF) [file pone.0196782.s007.pdf]

April 6, 2018

Brik R. Zivkovich
University of Colorado Denver
Department of Civil Engineering
Brik.zivkovich@ucdenver.edu

PLOS ONE:
Environmental Systems Research Institute, Inc. (Esri) grants to PLOS ONE a personal, nonexclusive, non-transferable, royalty-free, license and permission to use, copy, reproduce, redistribute, and publicly display the attached screenshot images contained in the file “fig3_revised” and “fig4_revised” (hereinafter the “Screenshots”) created using Esri® ArcGIS Online® for use in an upcoming journal article “Predicting nonpoint stormwater quality from land use” (hereinafter the “article”) to be published by PLOS ONE Under a CC_BY 4.0 open access license, in March/April 2018, to be made available in electronic versions with worldwide access. Esri reserves the right to grant permission for any other use of the Screenshots.
Use of the Screenshots is contingent upon proper copyright attribution being provided to Esri and its contributors. In the event that a user prepares a derivative work of the screenshots, the user shall remove the copyright attribution notice.

Used with permission. Copyright © 2018 Esri, ArcGIS Online, USGS, National Boundaries Dataset, 3DEP Elevation Program, Geographic Names Information System, National Hydrography Dataset, National Land Cover Database, National Structures Dataset, and National Transportation Dataset, U.S. Census Bureau - TIGER/Line and USFS Road **Data** and the GIS User Community.

Thank you for contacting Esri with your permission request. Please do not hesitate to contact me if I can be of further assistance.

Rhesha Moreau | IP Administrator
Contracts and Legal Department
Esri | 380 New York Street | Redlands, CA 92373 | USA
Phone: 909.793.2853 ext. 1-6238 |
email: rmoreau@esri.com | www.esri.com

380 New York Street
Redlands, California 92373-8100 USA

909 793 2853
info@esri.com

esri.com
