# Supplementary material for: Predicting nonpoint stormwater runoff quality from land use
Source: PLoS One. 2018 May 9;13(5):e0196782. doi: 10.1371/journal.pone.0196782 (PMC5942771; doi:10.1371/journal.pone.0196782)
Supplement: S2 File — The zip folder includes a read-me text file, the MATLAB code (as .m file), fourteen (14) supporting data files (as .txt for each constituent concentration and land use percentage), and a published MATLAB document (as .pdf) representing MATLAB’s run of the code using supporting data files and output of results from statistical analysis. (ZIP) [file pone.0196782.s008.zip › Zivkovich_PLOSONE_Matlab/crunch-publish.pdf]

```
function crunch(d)

% Function CRUNCH(d) performs the regression of stormwater quality versus
% land use. If d = 0, the default, it does not display the detailed
% statistics. If d = 1, then it does.

% PLOS ONE
% Title - Predicting nonpoint stormwater runoff quality from land use
% Authors - Brik R. Zivkovich, MS, EIT and David C. Mays, Ph.D., P.E.

% -------------------------------------------------------

if nargin == 0
    d = 0;
end

% load in concentration data and land use data for TSS
X_tss = load('1_TSS_Concentrations.txt');
LU_tss = load('1_TSS_LandUsePercentages.txt');
N_tss = length(X_tss);

% fit multiple linear regression model using FITLM
model_tss = fitlm(LU_tss,X_tss,'y ~ x1 + x2 + x3 - 1')

if d == 1
    % check statistics
    C_tss_res = X_tss(1:246);
    N_tss_res = length(C_tss_res);
    mean_tss_res = mean(C_tss_res)
    SE_tss_res = std(C_tss_res)/sqrt(N_tss_res)
    C_com_tss = X_tss(247:507);
    N_com_tss = length(C_com_tss);
    mean_com_tss = mean(C_com_tss)
    SE_com_tss = std(C_com_tss)/sqrt(N_com_tss)
    C_openspace_tss = X_tss(508:514);
    N_openspace_tss = length(C_openspace_tss);
    mean_openspace_tss = mean(C_openspace_tss)
    SE_openspace_tss = std(C_openspace_tss)/sqrt(N_openspace_tss)
end

% -------------------------------------------------------
% load in concentration data and land use data for TKN
X_tkn = load('2_TKN_Concentrations.txt');
LU_tkn = load('2_TKN_LandUsePercentages.txt');
N_tkn = length(X_tkn);

% fit multiple linear regression model using FITLM,
model_tkn = fitlm(LU_tkn,X_tkn,'y ~ x1 + x2 + x3 - 1')

if d == 1
    % check statistics
    C_res_tkn = X_tss(1:196);
    N_res_tkn = length(C_res_tkn);
    mean_res_tkn = mean(C_res_tkn);
    SE_res_tkn = std(C_res_tkn)/sqrt(N_res_tkn);
```

```
    C_com_tkn = X_tss(197:416);
    N_com_tkn = length(C_com_tkn);
    mean_com_tkn = mean(C_com_tkn);
    SE_com_tkn = std(C_com_tkn)/sqrt(N_com_tkn);
    C_openspace_tkn = X_tss(417:423);
    N_openspace_tkn = length(C_openspace_tkn);
    mean_openspace_tkn = mean(C_openspace_tkn);
    SE_openspace_tkn = std(C_openspace_tkn)/sqrt(N_openspace_tkn);
end

% -------------------------------------------------------
% load in concentration data and land use data for NO2+NO3
X_no2no3 = load('3_NO2NO3_Concentrations.txt');
LU_no2no3 = load('3_NO2NO3_LandUsePercentages.txt');
N_no2no3 = length(X_no2no3);

% fit multiple linear regression model using FITLM,
model_no2no3 = fitlm(LU_no2no3,X_no2no3,'y ~ x1 + x2 + x3 - 1')

if d == 1
    % check statistics
    C_res_no2no3 = X_tss(1:226);
    N_res_no2no3 = length(C_res_no2no3);
    mean_res_no2no3 = mean(C_res_no2no3);
    SE_res_no2no3 = std(C_res_no2no3)/sqrt(N_res_no2no3);
    C_com_no2no3 = X_tss(227:435);
    N_com_no2no3 = length(C_com_no2no3);
    mean_com_no2no3 = mean(C_com_no2no3);
    SE_com_no2no3 = std(C_com_no2no3)/sqrt(N_com_no2no3);
    C_openspace_no2no3 = X_tss(436:442);
    N_openspace_no2no3 = length(C_openspace_no2no3);
    mean_openspace_no2no3 = mean(C_openspace_no2no3);
    SE_openspace_no2no3 = std(C_openspace_no2no3)/sqrt(N_openspace_no2no3);
end

% -------------------------------------------------------
% load in concentration data and land use data for TP
X_tp = load('4_TP_Concentrations.txt');
LU_tp = load('4_TP_LandUsePercentages.txt');
N_tp = length(X_tp);

% fit multiple linear regression model using FITLM,
model_tp = fitlm(LU_tp,X_tp,'y ~ x1 + x2 + x3 - 1')

if d == 1
    % check statistics
    C_res_tp = X_tss(1:235);
    N_res_tp = length(C_res_tp);
    mean_res_tp = mean(C_res_tp);
    SE_res_tp = std(C_res_tp)/sqrt(N_res_tp);
    C_com_tp = X_tss(236:502);
    N_com_tp = length(C_com_tp);
    mean_com_tp = mean(C_com_tp);
    SE_com_tp = std(C_com_tp)/sqrt(N_com_tp);
    C_openspace_tp = X_tss(503:510);
    N_openspace_tp = length(C_openspace_tp);
    mean_openspace_tp = mean(C_openspace_tp);
```

```
    SE_openspace_tp = std(C_openspace_tp)/sqrt(N_openspace_tp);
end

%_________________________________________________________________________
% load in concentration data and land use data for DP
X_dp = load('5_DP_Concentrations.txt');
LU_dp = load('5_DP_LandUsePercentages.txt');
N_dp = length(X_dp);

% fit multiple linear regression model using FITLM,
model_dp = fitlm(LU_dp,X_dp,'y ~ x1 + x2 + x3 - 1')

if d == 1
    % check statistics
    C_res_dp = X_tss(1:192);
    N_res_dp = length(C_res_dp);
    mean_res_dp = mean(C_res_dp);
    SE_res_dp = std(C_res_dp)/sqrt(N_res_dp);
    C_com_dp = X_tss(193:364);
    N_com_dp = length(C_com_dp);
    mean_com_dp = mean(C_com_dp);
    SE_com_dp = std(C_com_dp)/sqrt(N_com_dp);
    C_openspace_dp = X_tss(365:371);
    N_openspace_dp = length(C_openspace_dp);
    mean_openspace_dp = mean(C_openspace_dp);
    SE_openspace_dp = std(C_openspace_dp)/sqrt(N_openspace_dp);
end

%_________________________________________________________________________
% load in concentration data and land use data for Cu
X_cu = load('6_Cu_Concentrations.txt');
LU_cu = load('6_Cu_LandUsePercentages.txt');
N_cu = length(X_cu);

% fit multiple linear regression model using FITLM,
model_cu = fitlm(LU_cu,X_cu,'y ~ x1 + x2 + x3 - 1')

if d == 1
    % check statistics
    C_res_cu = X_tss(1:186);
    N_res_cu = length(C_res_cu);
    mean_res_cu = mean(C_res_cu);
    SE_res_cu = std(C_res_cu)/sqrt(N_res_cu);
    C_com_cu = X_tss(187:271);
    N_com_cu = length(C_com_cu);
    mean_com_cu = mean(C_com_cu);
    SE_com_cu = std(C_com_cu)/sqrt(N_com_cu);
    C_openspace_cu = X_tss(272:278);
    N_openspace_cu = length(C_openspace_cu);
    mean_openspace_cu = mean(C_openspace_cu);
    SE_openspace_cu = std(C_openspace_cu)/sqrt(N_openspace_cu);
end

%_________________________________________________________________________
% load in concentration data and land use data for Zn
X_Zn = load('7_Zn_Concentrations.txt');
LU_Zn = load('7_Zn_LandUsePercentages.txt');
```

```
N_Zn = length(X_Zn);

% fit multiple linear regression model using FITLM,
model_Zn = fitlm(LU_Zn,X_Zn,'y ~ x1 + x2 + x3 - 1')

if d == 1
    % check statistics
    C_res_Zn = X_tss(1:155);
    N_res_Zn = length(C_res_Zn);
    mean_res_Zn = mean(C_res_Zn);
    SE_res_Zn = std(C_res_Zn)/sqrt(N_res_Zn);
    C_com_Zn = X_tss(156:238);
    N_com_Zn = length(C_com_Zn);
    mean_com_Zn = mean(C_com_Zn);
    SE_com_Zn = std(C_com_Zn)/sqrt(N_com_Zn);
    C_openspace_Zn = X_tss(239:245);
    N_openspace_Zn = length(C_openspace_Zn);
    mean_openspace_Zn = mean(C_openspace_Zn);
    SE_openspace_Zn = std(C_openspace_Zn)/sqrt(N_openspace_Zn);
end
```

```
model_tss = 


Linear regression model:
    y ~ x1 + x2 + x3

Estimated Coefficients:
          Estimate      SE       tStat       pValue  
          ________    ______    ______    __________

    x1     204.24      18.53    11.022    1.7152e-25
    x2     193.41      17.99    10.751    1.9179e-24
    x3     396.72     109.85    3.6115     0.0003345


Number of observations: 514, Error degrees of freedom: 511
Root Mean Squared Error: 291

model_tkn = 


Linear regression model:
    y ~ x1 + x2 + x3

Estimated Coefficients:
          Estimate      SE        tStat       pValue  
          ________    _______    ______    __________

    x1     3.4064     0.17119    19.899    1.5097e-62
    x2     2.5341     0.16158    15.683    5.7899e-44
    x3     2.8843     0.90584    3.1841     0.0015602


Number of observations: 423, Error degrees of freedom: 420
```

```
Root Mean Squared Error: 2.4

model_no2no3 =


Linear regression model:
    y ~ x1 + x2 + x3

Estimated Coefficients:
          Estimate       SE        tStat       pValue  
          ________    ________    ______    __________

    x1     1.0696     0.047943     22.31    2.9455e-74
    x2    0.70498     0.049855    14.141    1.1245e-37
    x3    0.51571      0.27242    1.8931      0.059001


Number of observations: 442, Error degrees of freedom: 439
Root Mean Squared Error: 0.721

model_tp =


Linear regression model:
    y ~ x1 + x2 + x3

Estimated Coefficients:
          Estimate       SE        tStat       pValue  
          ________    ________    ______    __________

    x1    0.51136     0.023281    21.965    1.3512e-75
    x2    0.27677     0.021841    12.672     3.735e-32
    x3    0.41286      0.13489    3.0607     0.0023258


Number of observations: 509, Error degrees of freedom: 506
Root Mean Squared Error: 0.357

model_dp =


Linear regression model:
    y ~ x1 + x2 + x3

Estimated Coefficients:
          Estimate       SE        tStat       pValue  
          ________    ________    ______    __________

    x1     0.24885    0.013795     18.04    1.4193e-52
    x2    0.089302    0.014575    6.1273    2.3002e-09
    x3     0.13429    0.072246    1.8587      0.063862


Number of observations: 371, Error degrees of freedom: 368
Root Mean Squared Error: 0.191

model_cu =
```

```
Linear regression model:
    y ~ x1 + x2 + x3

Estimated Coefficients:
          Estimate      SE      tStat       pValue
          ________    ______    ______    __________

    x1    20.089      1.8365    10.939    2.3228e-23
    x2    27.585      2.7167    10.154    8.8702e-21
    x3    37.143      9.4669    3.9234    0.00011034


Number of observations: 278, Error degrees of freedom: 275
Root Mean Squared Error: 25

model_Zn =


Linear regression model:
    y ~ x1 + x2 + x3

Estimated Coefficients:
          Estimate      SE      tStat       pValue
          ________    ______    ______    __________

    x1    104.01      11.593    8.9713    8.2755e-17
    x2    143.14      15.843    9.0348    5.3664e-17
    x3    101.43      54.554    1.8592      0.064209


Number of observations: 245, Error degrees of freedom: 242
Root Mean Squared Error: 144
```
